# Light and Temperature Shape the Phenylpropanoid Profile of *Azolla filiculoides* Fronds

**DOI:** 10.3389/fpls.2021.727667

**Published:** 2021-10-21

**Authors:** Alma Costarelli, Sara Cannavò, Martina Cerri, Roberto Maria Pellegrino, Lara Reale, Francesco Paolocci, Stefania Pasqualini

**Affiliations:** ^1^Department of Chemistry, Biology and Biotechnology, University of Perugia, Perugia, Italy; ^2^Department of Agricultural, Food and Environmental Sciences, University of Perugia, Perugia, Italy; ^3^Institute of Bioscience and Bioresources (IBBR), National Research Council (CNR), Perugia, Italy

**Keywords:** *Azolla filiculoides*, cold stress, gene expression, liquid chromatography coupled with high resolution mass spectrometry (LC/HRMS), metabolic profile, polyphenols

## Abstract

*Azolla* is a genus of floating freshwater ferns. By their high growth and N_2_ fixation rates, *Azolla* species have been exploited for centuries by populations of South-east Asia as biofertilizers in rice paddies. The use of *Azolla* species as a sustainable plant material for diverse applications, such as feeding, biofuel production, and bioremediation, has encountered a growing interest over the last few years. However, high levels of feed deterrent flavonoids in their fronds have discouraged the use of these ferns as a sustainable protein source for animal consumption. Additionally, information on how and to what extent environmental determinants affect the accumulation of secondary metabolites in these organisms remains poorly understood. Moving from these considerations, here, we investigated by an untargeted metabolomics approach the profiles of phenylpropanoid compounds in the fronds of *Azolla filiculoides* sampled under control and pigment-inducing stress conditions. In parallel, we assayed the expression of essential structural genes of the phenylpropanoid pathway by quantitative RT-PCR. This study provides novel information concerning *A. filiculoides* phenylpropanoid compounds and their temporal profiling in response to environmental stimuli. In particular, we show that besides the already known 3-deoxyanthocyanidins, anthocyanidins, and proanthocyanidins, this fern can accumulate additional secondary metabolites of outstanding importance, such as chemoattractants, defense compounds, and reactive oxygen species (ROS) scavengers, and crucial as dietary components for humans, such as dihydrochalcones, stilbenes, isoflavones, and phlobaphenes. The findings of this study open an opportunity for future research studies to unveil the interplay between genetic and environmental determinants underlying the elicitation of the secondary metabolites in ferns and exploit these organisms as sustainable sources of beneficial metabolites for human health.

## Introduction

*Azolla* is a genus of floating freshwater ferns that encompasses seven species, distributed from tropical to temperate zones in the world (Wagner, 1997). Thanks to the symbiosis with *Trichormus azollae*, a nitrogen-fixing species of bacteria and, likely, several other helpful microorganisms colonizing their leaf cavities, these species can double their biomass in a short period of time. The *Azolla*/*Trichormus azollae* symbiosis likely arose 90 million years ago (Metzgar et al., 2007), before legume/rhizobia symbioses that, in contrast, evolved about 64 million years ago (Herendeen et al., 1999). However, when compared with other plant prokaryotic symbioses, the *Azolla/Trichormus azollae* symbiosis is not only the one to persist throughout the reproductive cycle of a host plant, being the bacterium transmitted vertically during the life cycle of ferns, but also among the most efficient ones in terms of N_2_ fixation ability (Ran et al., 2010). By their high growth and N_2_ fixation rates, *Azolla* spp. has been exploited for centuries by populations of Southeast Asia as biofertilizers in rice paddies (Lumpkin and Plucknett, 1980). Recent research has highlighted their potential to source proteins for animal nutrition (Brouwer et al., 2018). In this context, outstanding features, such as amino acid profiles of their proteins, close to that of soybean, the crop that most largely contributes to feeding livestock, coupled to low-to-null input-demanding farming systems, have sparked interest in these species (Brouwer et al., 2017).

Notwithstanding, the digestibility and bioassimilability of fern forage can be seriously compromised by the qualitative and quantitative profiles of secondary metabolites accumulated by these species because of changing environmental conditions. Although 3-deoxyanthocyanins have been long regarded as the sole flavonoids made by *Azolla* spp. (Ishikura, 1982; Cohen et al., 2002a,b), new evidence suggests that proanthocyanidins (PAs) are the flavonoids that most likely prominently affect *Azolla* spp. palatability (Brouwer et al., 2018). The presence, composition, and extent of polymerization are well-documented features that control the ability of PAs to bind forage proteins and, hence, dictate forage palatability and the extent to which plant proteins are converted into animal proteins by livestock (Mueller-Harvey et al., 2019).

Flavonoids are derived from the phenylpropanoid pathway and are among the most abundant groups of secondary plant metabolites. These metabolites exert several beneficial properties in plants spanning from communication with helpful (micro)organisms down to defense against biotic and abiotic stress (Dixon and Paiva, 1995; Hernández et al., 2009; Petrussa et al., 2013; Davies et al., 2018). Albeit with a few exceptions, major classes of flavonoid biosynthesis have been well-characterized in major crops and plant model spp. The process starts from phenylalanine *via* reactions catalyzed by phenylalanine ammonia-lyase (PAL), cinnamate 4-hydroxylase (C4H), 4-coumarate: CoA ligase (4CL), chalcone synthase (CHS), and chalcone isomerase (CHI) to produce flavanones. In turn, flavanones are substrates for the biosynthesis of various classes of flavonoids, namely, flavones, flavonols, isoflavanols, anthocyanins, flavan-4-ols, 3-deoxyanthocyaininds, phlobaphenes, flavan-3-ols, and PAs. From flavanones, the pathways of blue-red pigment anthocyanins and those of colorless flavan-3-ols diverge from brick-red pigment 3-deoxyanthocyanins (Hipskin et al., 1996). For the biosynthesis of the first two classes of pigments, flavanones are catalyzed by flavanone-3-hydroxylase (F3H) to dihydroflavonols, which are then converted to anthocyanins and/or proanthocyanidins by a series of enzymatic activities, the first of which is catalyzed by dihydroflavonol 4-reductase (DFR). Conversely, 3-deoxyanthocyanins likely derive from the direct reduction of flavanones by DFR or the DFR-like enzyme flavanone 4-reductase (FNR) to give the critical intermediate flavan-4-ols, apiforol and luteoforol (Wharton and Nicholson, 2000; Chopra et al., 2002), which are then dehydrogenated/dehydroxylated by the action of an ANS-like enzyme (Liu et al., 2010). The biosynthesis of phlobaphenes, polymers of flavan-4-ols that accumulate in several plant species, such as maize and sorghum, remains uncharacterized as well.

If the high accumulation of flavonoids represents the detrimental issue that prevents the use of *Azolla* species as a sustainable source of proteins for animal consumption, it might represent an opportunity to be pursued to isolate and exploit beneficial metabolites for human diets from these organisms. To the best of our knowledge, this avenue remains to be followed. Several studies have addressed the metabolic changes triggered by stresses in these species (Dai et al., 2012; Brouwer et al., 2017; Nham Tran et al., 2020; Güngör et al., 2021). However, information on how and to what extent environmental determinants affect the accumulation of secondary metabolites in ferns remain scanty and fragmented. To this end, here, we embarked on a study addressed to shed more light on the role of temperature and light in shaping the overall accumulation of phenylpropanoids, and flavonoids in particular, in *Azolla filiculoides*. To reach this goal, we initially performed a targeted expression profile analysis by qRT-PCR of the phenylpropanoid pathway's candidate key structural genes. Simultaneously, we performed an untargeted high-throughput metabolomics analysis through LC/HRMS on reddish *Azolla* harvested in Winter at the Botanical garden of Perugia University and upon its acclimatization in a climatic chamber. Under the latter condition, Azolla displayed a green color. This approach gave us an overview of the secondary metabolites accumulated by this organism under two different environmental conditions. Then, metabolic and molecular changes were monitored in the same specimen over 20 days of treatment under moderate light intensity- and cold-controlled conditions.

This study provides novel information concerning *A. filiculoides* phenylpropanoid compounds and their temporal profiling in response to environmental stimuli. The plasticity and vast array of secondary metabolites detected here, coupled to its amenability to grow under limiting conditions, and the availability of its complete genome (Li et al., 2018), make *A. filiculoides* a potentially innovative source of useful secondary metabolites and model among ferns to unearth their regulation.

## Materials and Methods

### Plant Materials and Growing Conditions

#### Plant Materials

The specimens of *Azolla* employed in this study were collected in February 2020 from a small pond of the botanical garden of the University of Perugia–Italy [latitude 43°05′52″N, longitude 12°23′49″E (maximum, minimum, and mean monthly temperatures of this location are given in Supplementary Figure S1) and day length of about 10.5 h]. Sampling was performed in six replicates from different sites of the pond by collecting ferns from about 10 cm^2^ of pond area per replicate. All the samples (BG samples thereafter) showed a reddish phenotype. At sampling, part of the fresh material was moved to a controlled growing condition as reported below, part was used for microscopic examination and part was frozen in liquid nitrogen and then stored at −80°C for subsequent biochemical and molecular analyses. The sampling of material was always performed between 9:30 and 10:30 a.m. (i.e., 1.5–2.5 h into the 10-h light photoperiod for samples grown under controlled conditions) to minimize bias due to diel transcriptomic changes.

#### Seasonal Temperature and Light Intensity at Sampling Site

The data of seasonal temperature changes in Perugia during the period October 2019–February were provided by the Hydrographic Service of the Regione Umbria (https://www.regione.umbria.it/ambiente/servizio-idrografico). The monthly means of global solar radiation during the same period were provided by the ARPA Umbria (http://www.arpa.umbria.it).

#### Growing Conditions of *Azolla* Specimen in Climatic Chamber

The BG samples were moved to a climatic chamber with a 10-h photoperiod, 25/20°C day/night temperature, and a photosynthetic photon flux density (PPFD) of 220 μmoles m^−2^ s^−1^ provided by fluorescent tubes, and grown in 50-cm (length) × 33-cm (width) × 11-cm (depth) tanks filled with a 6-L nitrogen-free nutrient medium (Watanabe et al., 1992). The samples grown in the climatic chamber under the conditions above from now on will be referred to as CC. The nutrient medium was changed every week, senescent plant material was removed manually, and fresh cultures made up of young plants were established every 2–4 weeks for each of the six samples originally collected from the pond. After further 9 months of cultivation as described above, *Azolla* specimens were employed to test for environmental conditions that might trigger the reddish color of the fronds. To this end, three tanks, set up as above, were filled with *Azolla* plants, each with young plants from a different batch, and the temperature and PPFD were changed to 5°C and 700, respectively. The sampling of plants was carried out at the onset of the experiment (T_0_) and after 24 (T1d), 48 (T2d), and 72h (T3d), and 20 days (T20d) of cold treatment. The entire experimental set was repeated twice. The ferns sampled at different time points under stress-induced conditions, as well as the CC samples, were washed, their roots were removed, and the fronds were frozen in liquid N_2_ and stored at −80°C for further biochemical and molecular analyses.

### Morphological and Molecular Analyses of *Azolla* Specimen

#### Light Microscopy and PA Staining

The *Azolla* fronds were fixed in 3% (w/v) glutaraldehyde in 0.075-M phosphate buffer, pH 7.2, for 24 h. Each of the samples were then washed three times for 7 min in the 0.075-M phosphate buffer, pH 7.2, and post-fixed in 1% (w/v) OsO_4_ for 1.5 h. The samples were then dehydrated in increasing ethanol concentrations and embedded in resin (Epon, 2-dodecenylsuccinic anhydride, and methylnadic anhydride mixture). Semi-thin sections (1–2 μm) were cut with an ultramicrotome (OmU2; Reichert, Heidelberg, Germany) equipped with a glass blade, stained with toluidine blue, and observed using a light microscope (BX53; Olympus, Tokyo, Japan) (Reale et al., 2017). Red *Azolla* fronds taken from the botanical garden were free-hand sectioned using a razor blade. They were then mounted on slide glasses with a drop of distilled water and examined in a bright field using the light microscope. The staining of flavan-3-ols and PAs with 4-dimethylaminocinnamaldehyde (DMACA) (Merck Life Science, Milan, Italy) was basically performed as reported in Abeynayake et al. (2011).

#### DNA Isolation, Amplification, and Sequencing of PCR Products

Deoxribonucleic acid was isolated from the *Azolla* fronds according to Doyle and Doyle (1987). Internal transcribed spacer (ITS) sequences of nuclear ribosomal DNA (nrDNA) of *Azolla* spp. were downloaded from Genbank and Fernbase (https://www.fernbase.org). The latter database reports the whole genome of *Azolla filiculoides*. Sequence alignment was carried out using BioEdit (version 7.2.5) and, by taking into account most conserved ITS regions, a forward primer on the 3′ end of the small rDNA subunit (5′-cctgcggaaggatcattgacacac); forward (5′-gaattccgcgaatcatcgagt) and reverse (5′-cgttcttcatcgttgcaagagcc) primers on the 5.8S gene and a reverse (5′-tctcgcctgatctgaggtccgtt) primer on the 5′ end of the large rDNA subunit of *Azolla* spp. were designed using OligoExpress (version 2.0) (Applied Biosystems, Waltham, MA, USA). These primers were employed in different combinations to amplify *Azolla* DNA using the polymerase Phusion High-Fidelity DNA (Thermo Fisher Scientific, Milan, Italy) in a 9700 GeneAmp (Applied Biosystems, Waltham, MA, USA) apparatus. PCR products were visualized on 1% agarose gels stained with ethidium bromide, purified, and double-strand sequenced using gene-specific primers and the Big Dye Terminator Cycle Sequencing Kit according to the instructions of the supplier. The sequencing reactions were run on an ABI Prism 310 Sequence Analyzer (Applied Biosystems, Waltham MA, USA).

### Isolation and Quantification of Phenolic Compounds in *Azolla filiculoides* Fronds

#### Spectrophotometric Analysis

Anthocyanidins and deoxyanthocyanidins were extracted as described by Dong et al. (2001), with slight modifications. One hundred mg of *Azolla* fronds were extracted with liquid nitrogen in 1.5 ml of 1% (v/v) HCl in methanol and left at 4°C for overnight agitation. The extract was then centrifuged at 13,000 rpm at 4°C for 10 min, and 1 ml of distilled water was added to the supernatant. Chlorophyll was separated by back extraction with 1.5 ml of chloroform. The anthocyanidins were quantified using A_530_ for cyanidin 3-glucoside (ε = 26,900 L m^−1^mol^−1^). For deoxyantocyanidin quantification, the extract obtained as above was read at 479 nm for apigeninidin (ε = 38,000 L m^−1^mol^−1^) and 496 nm for luteolinidin-5-*O*-glucoside, as reported in Cohen et al. (2002a).

Spectrophotometric determination of phenolic acids and flavonols was carried out following Cassani et al. (2017), with slight modifications. Fifteen mg of *Azolla* fronds was first boiled with 200 μl of distilled water for 30 min and then left at 4°C for overnight agitation with 1.5 ml of an extraction solution (1% HCl, 95% ethanol). The extract was then centrifuged at 13,000 rpm at 4°C for 10 min, and the supernatant was read at 350 nm for flavonols and at 280 nm for phenolic acids. The amount of flavonols was calculated as quercetin 3-glucoside equivalents (ε = 21,877 L m^−1^mol^−1^, MW 464.82) and the amount of phenolics was calculated as ferulic acid equivalents (ε = 14,700 L m^−1^mol^−1^MW 194.18).

Phlobaphenes were extracted from 100 mg of *Azolla* fronds with 200 μl of concentrated HCl and 800 μl of dimethyl sulfoxide (DMSO) added sequentially with vigorous vortexing after each addition. Extracts were then centrifuged at 14,000 rpm at 4°C for 45 min, and cleared supernatants were diluted with methanol (20% final concentration). Phlobaphene concentration was expressed as absorbance value at their λmax (510 nm) per 1 g of fresh weight, as Landoni et al. (2020) reported.

For the quantification of both soluble and insoluble Pas, 100 mg of *Azolla* fronds were extracted and quantified, as reported in Li et al. (1996).

#### Liquid Chromatography Coupled With High-Resolution Mass Spectrometry (LC/HRMSLC/HRMS) Analysis

The total phenolic compounds were extracted as reported by Jiang et al. (2013), with minor modifications. A 100 mg sample was ground in liquid nitrogen and extracted with a 1 ml extraction solution (80% methanol:1% HCl) using mortar and pestle. After centrifugation at 4,000 *g* for 15 min, the residues were re-extracted twice as described above, and the supernatants were filtered through a 0.22 μm membrane.

The extracts were analyzed by liquid Chromatography coupled with high resolution mass spectrometry (LC/HRMS). In this regard, the instrumental conditions for analyzing phenolic compounds were optimized previously (Qian et al., 2020). Briefly, a UHPLC analysis was performed using an Agilent 1260 UHPLC (Agilent Technology, Santa Clara, CA, USA) system equipped with an autosampler with a thermostated column compartment and a quaternary pump.

A chromatographic separation was performed on an InfinityLab Poposhell 120 EC-C8 column (2.1 mm × 100 mm, 1.9 μm; Agilent Technology, Santa Clara, CA, USA). The mobile phase consisted of water (A) and acetonitrile (B), with both containing 0.1% of acetic acid, and was delivered at a flow rate of 0.30 ml/min under the following gradient procedure: 0–7 min, 5–40% B; 7–8 min, 40–98% B; Column temperature was 50°C, and injection volume was 3 μl. All the samples were kept at 20°C during the analysis.

An MS detection was performed on an Agilent 6530 (Agilent Technology, Santa Clara, CA, USA) accurate mass quadrupole time of flight (QTOF) system equipped with an Agilent Jet Stream (Agilent Technology, Santa Clara, CA, USA) source. The source operated in both polarities as follows: ion spray voltage, 3,500 V; gas temp and sheath gas temp were set at 250 and 300 °C, respectively; nebulizer (N2) 35 psi; sheath gas flow 12 L/min. Data-dependent acquisition was used in the mass range of 50–17,00 m/z both for MS and MS/MS analyses. Raw data were processed with MS-DIAL (Tsugawa et al., 2015) to perform peak detection, integration, alignment, and metabolite annotation. The annotation was based on a comparison of spectrometric data with those from the NIST2020 tandem mass library. Valid scores >0% and mass differences <15 ppm were considered. Then, the annotation list provided by the library was curated manually to select, among the proposed isobaric metabolites, the most likely ones according to our experience and literature. The two sets of data obtained, one for each polarity, were merged into a single table for subsequent statistical and chemometric analyses. Among the metabolites present in both polarities, those with a larger mean area were selected for the analysis. The annotation data of the selected metabolites are given in Supplementary Table S1.

### Gene Expression Analysis

#### RNA Isolation and cDNA Synthesis

Ribonucleic acid was isolated from 50 mg of *Azolla* fronds with the Spectrum Plant Total RNA Kit (Sigma–Aldrich, Milan, Italy) applying protocol B, and then treated with DNase (Sigma–Aldrich, Milan, Italy) according to the instructions of the supplier. The null PCR amplification in the presence of *Azolla*-specific ITS primers reported above verified the absence of any DNA contaminating the RNA preparations. According to the instructions of the supplier, 3 μg of RNA was reverse-transcribed in the presence of Maxima H Minus Reverse Transcriptase (Thermo Fisher Scientific, Milan, Italy) and 100 pmol of random hexamers (Euroclone, Milan, Italy).

#### Identification of Candidate Genes of Phenylpropanoid Pathway and Quantitative RT-PCR

Key structural genes of the phenylpropanoid pathway in *A. filiculoides* were searched in the *Azolla filiculoides* protein v1.1. database using the sequences of experimentally validated enzymes from model and crop plant species as a query in BLAST P. Once retrieved, the sequences of candidate genes were aligned to those of reference enzymes and phylogenetic trees built with MEGA -X using default parameters. At the same time, a study was in progress (Güngör et al., 2021), and Piatkowski et al. (2020) produced a list of candidate *A. filoculoides* phenylpropanoid genes; thus, the genes identified here were named according to the two previous studies. Gene-specific and/or family-specific primer pairs were then designed for qRT-PCR analyses with the help of OligoExpress Software (Applied Biosystems, Whaltham, MA, USA). The genes considered in this study and their relative primer pairs are given in Supplementary Table S2. An aliquot, which was made using the BlasTaq 2X qPCR Mater Mix (ABM, Richmond, Canada), of 3 μl of 1:10 diluted cDNA was used in the PCR and run into an ABI PRISM 7300 SDS apparatus (Applied Biosystems, Waltham, MA, USA) using the following cycling parameters: an initial step at 95°C for 3 min, then 40 cycles each including a step at 95°C for 15 s and a step at 60°C for 1 min. A melting curve was added after each run. For each gene, four technical replicates were amplified. The efficiency of PCR for each primer pair was tested as reported in Escaray et al. (2017) before the (2^−Δ*Ct*^) gene expression quantification method was applied based on the differences between the relative expression of the target genes and the reference *EF-1a* gene to compare the relative expression profiles among genes and treatments as reported in Bizzarri et al. (2020). Four biological replicates for each treatment were analyzed; the significance of the expression values was analyzed by a two-way ANOVA (*p* < 0.05) procedure embedded in the R statistical package.

### Statistical Analysis

The different experiments were repeated twice with three replicates each. Statistical analyses were carried out in MetaboAnalyst 5.0 (Pang et al., 2021) and/or R Studio (version 3.5.3). To determine significant differences between the treatments, the data were subjected to an unpaired two-sample Student's *t*-test (*P* < 0.05, FDR = 0.05) or ANOVA (*P* < 0.001, FDR = 0.001) followed by *post-hoc* comparison [Tukey's honestly significant difference (HSD), *P* < 0.05].

Multivariate statistics allowed for the computation of heat maps and PCAs after data normalization by median and Pareto-scaling (mean-centered and divided by the square root of the standard deviation of each variable).

## Results

### Features and Identification of *Azolla* Specimen Grown in the Botanic Garden of Perugia University

The BG samples collected in February 2020 showed a bright red color (Supplementary Figure S2A). Light microphotographs of hand-made cross-sections revealed epidermal cells completely filled with red pigments (Supplementary Figure S2B). In keeping with the observation that leaf pigmentation is mainly influenced by low temperatures (Hughes et al., 2005), the minimum temperatures recorded at the collection site during the period October 2019–February 2020 ranged from 4 to 13°C (Supplementary Figure S1A). At the time of collection, the global solar radiation in the area was around 600 PPFD (Supplementary Figure S1B). The observation that areas of the pond shaded by the vegetation above the fronds were green or greenish (Supplementary Figure S3) prompted us to speculate that the red pigmentation of *Azolla* leaves requires both low temperatures and full sunlight. By this, <30 days of acclimation to climatic chamber conditions (temperatures of 25/20°C day/night, 10-/14-h light/dark, 220 PPFD) were sufficient to make the color of the ferns return to bright green (Supplementary Figure S2C).

**Figure 1:**
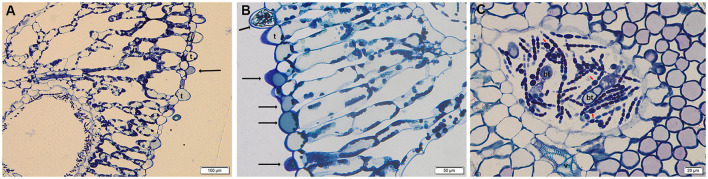
Semithin transversal section of *Azolla* fronds stained with toluidine blue. **(A,B)** Single cell trichomes (t) in the leaf upper lobes; phenolic compounds are highlighted by black arrows in epidermal cells and trichomes. **(C)** Philamentous bacterium *Trichormus* in the leaf cavity, with some nitrogen-fixing heterocystis (red arrows); bicellular trichomes (bt), and vessels (v) are evident.

The morphology of leaf trichomes, features of glochidia, and perine structure of the megaspore are likely the most reliable morphological traits to type among *Azolla* spp. (Perkins et al., 1985; Zimmerman et al., 1989). The trichomes on the surface of the leaflet upper lobes of *Azolla* sampled at the botanical garden were unicellular, a feature that typifies *A. filiculoides* (Pieterse et al., 1977), and they resulted from the protuberance of single epidermal cells (Figure 1A). The staining with toluidine blue highlighted blue phenolic deposits (Gutmann, 1995) in almost all the unicellular epidermal trichomes (Figures 1A,B), whereas DMACA staining evidenced the presence of PAs in the epidermal cells and unicellular trichomes both in the BG and growth chamber samples (not shown). An extracellular leaf cavity, constituted by infolding of the adaxial epidermis during development and housing hosted filamentous *Trichormus* bacteria, was also observed (Figure 1C) (Peters and Meeks, 1989). Unlike epidermal trichomes, different kinds of bicellular trichomes in the extracellular cavity were evident: simple hairs, primary and secondary branched hairs (Figure 1C) (Calvert and Peters, 1981). These trichomes stained with DMACA reinforce the observation that they are competent to accumulate PAs (Pereira and Carrapico, 2007).

For the identification of the plant material collected by molecular markers, ITS sequences from *Azolla* spp. present in National Center for Biotechnology Information (NCBI) were retrieved and aligned to design *Azolla*-specific ITS primers. These primers were employed to amplify and sequence the DNA isolated from the BG samples. The sequencing of resulting amplicons revealed the presence of both insertions and deletions either in the ITS1 or ITS2 regions specific to *A. filiculoides*, and an identity ranging from 97.21 to 100% with *A. filiculoides* sequences present in NCBI (Supplementary Figure S4). Therefore, based on morphological and molecular analyses, the *Azolla* BG specimen was classified as *A. filiculoides* Lam.

### Profiling of Polyphenols From BG and CC *A. filiculoides* Samples

Cold induces the accumulation of deoxyanthocyanins (Pieterse et al., 1977), with the red pigment luteolinidin 5-*O*-glucoside being recognized as a stable taxonomic chemical marker of the genus *Azolla* (Iwashina et al., 2010). As the first point of embarkation to determine the accumulation patterns of phenylpropanoids in *A. filiculoides*, we carried out a spectrophotometric analysis to compare the metabolic profiles of the reddish BG with the greenish CC ferns (Table 1). Higher levels of soluble PAs (almost 2-fold), insoluble PAs (up to 1.7-fold), anthocyanidins (up to 5-fold), and flavonols (up to 3.6-fold) were recorded in the BG samples. These samples also showed higher levels of deoxyanthocyanins apigeninidin (up to 9.2-fold) and luteolinidin 5-*O*-glucoside (up 9.6-fold) with respect to the CC samples. Interestingly, a constitutive accumulation of phlobafenes, which is significantly higher (up 2.2-fold) in the BG samples, was recorded in this fern. Phenolic acids appeared to be the most abundant polyphenolic component, but their content was not higher in the BG ferns.

**Table 1:** Polyphenol signature detected by spectrophotometric analyses in *A. filiculoides* sampled outdoor in February 2020 as compared with the fern grown in the climatic chamber (220 PPFD of light intensity; 10-h photoperiod, 25/20°C day/night temperature).

	**Anthocyanidins (nmol/gFW)**	**Phlobaphenes (Abs_**510**_/gFW)**	**Flavonols (nmol/gFW)**	**Phenolic acids (nmol/gFW)**	**Apigeninidin (nmol/gFW)**	**Luteolinidin-5-*O* glucoside (Abs_**496**_/gFW)**	**Soluble proanthocyanidins (mg catechin/gFW)**	**Insoluble proanthocyanidins (nmol/gFW)**
Climatic chamber	26.97 ± 6.65	7.35 ± 1.26	876.89 ± 76.73	2128.72 ± 199.31	36.28 ± 7.51	0.93 ± 0.21	504.93 ± 83.30	453.53 ± 44.47
Botanical garden	136.16 ± 16.41^***^	16.57 ± 1.24^***^	3187.62 ± 565.79^***^	2411.44 ± 211.91	333.07 ± 9.50^***^	8.91 ± 0.34^***^	990.64 ± 171.01^*^	787.83 ± 130.00^*^

*Results are means ± standard error (SE) of six biological replicates. Significant differences determined by t-test are indicated as*

*** *p < 0.001 and*

* *p < 0.05*.

An untargeted metabolomic approach based on liquid chromatography coupled with high-resolution mass spectrometry (LC/HRMS) was then employed to get a more in-depth profile of the secondary metabolites and allowed us to putatively annotate more than 140 phenolic compounds based on molecular mass and mass spectrum using the NIST2020 Tandem Mass Spectral Library (Supplementary Table S3). First, the untargeted chemometric technique of PCA was employed to have a first exploratory investigation of the data obtained. Using normalized peak areas and PCA clustering of all annotated polyphenols, the two groups of samples showed clear differences from each other (Supplementary Figure S5), with the first component explaining 64% of the sample variance. Specifically, the CC samples were clearly separated from the BG samples along PC1, indicating that they had diverse metabolic profiles. On the contrary, the residual 12% of variance explained by PC2 did not divide the samples into two groups.

**Figure 2:**
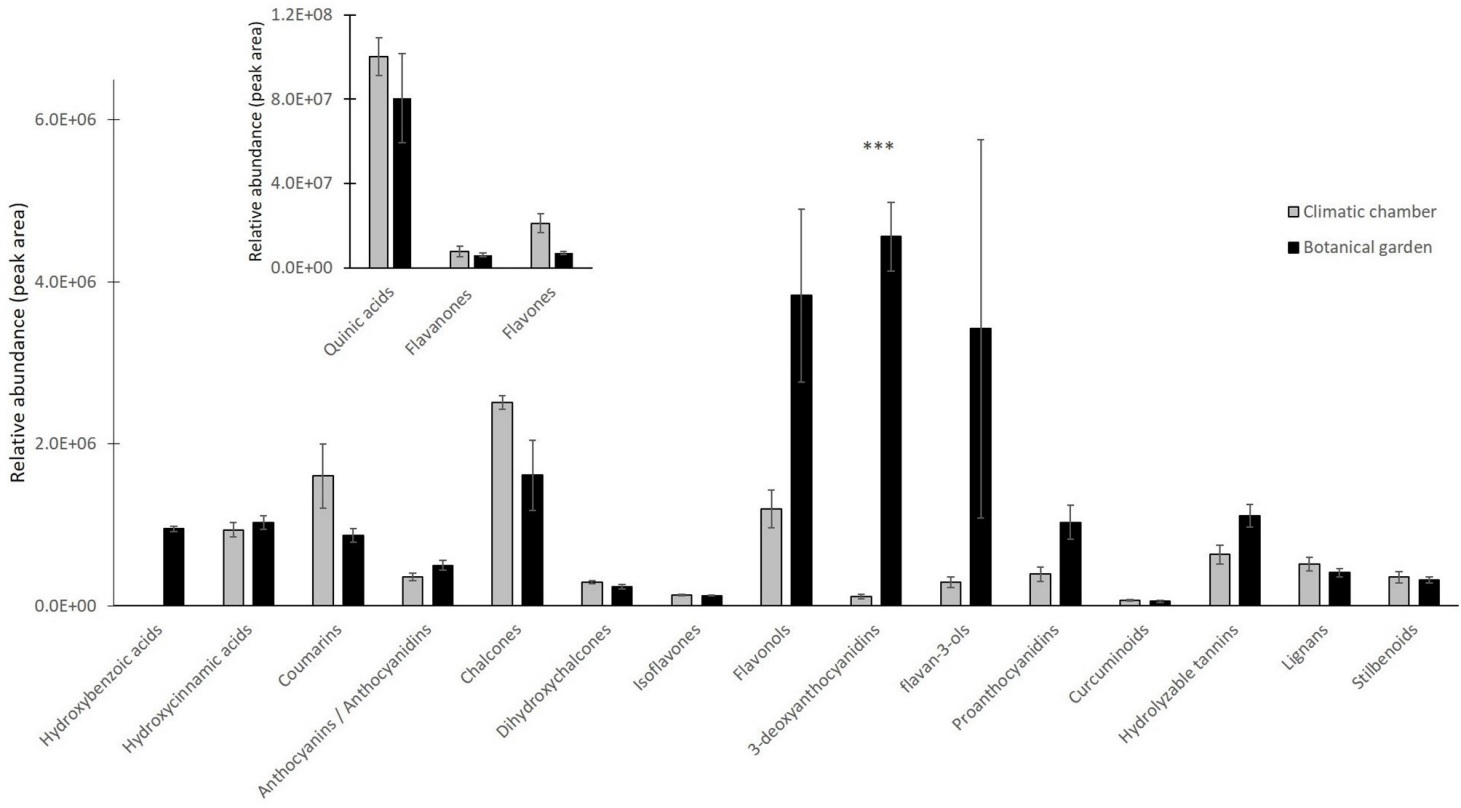
Relative abundance of different subclasses of polyphenols detected by liquid chromatography coupled with high resolution mass spectrometry (LC/HRMS) analysis in CC (220 photosynthetic photon flux density (PPFD) of light intensity; 10-h photoperiod, 25/20°C day/night temperature) vs. BG samples. Quinic acid, flavanones, and flavones were reported in a different framed histogram with higher y-axis scale for better visualization. Data are mean ± standard error (SE) of six biological replicates. Asterisks indicate significant differences between treatments as determined by *t*-test for *P* < 0.001.

**Figure 3:**
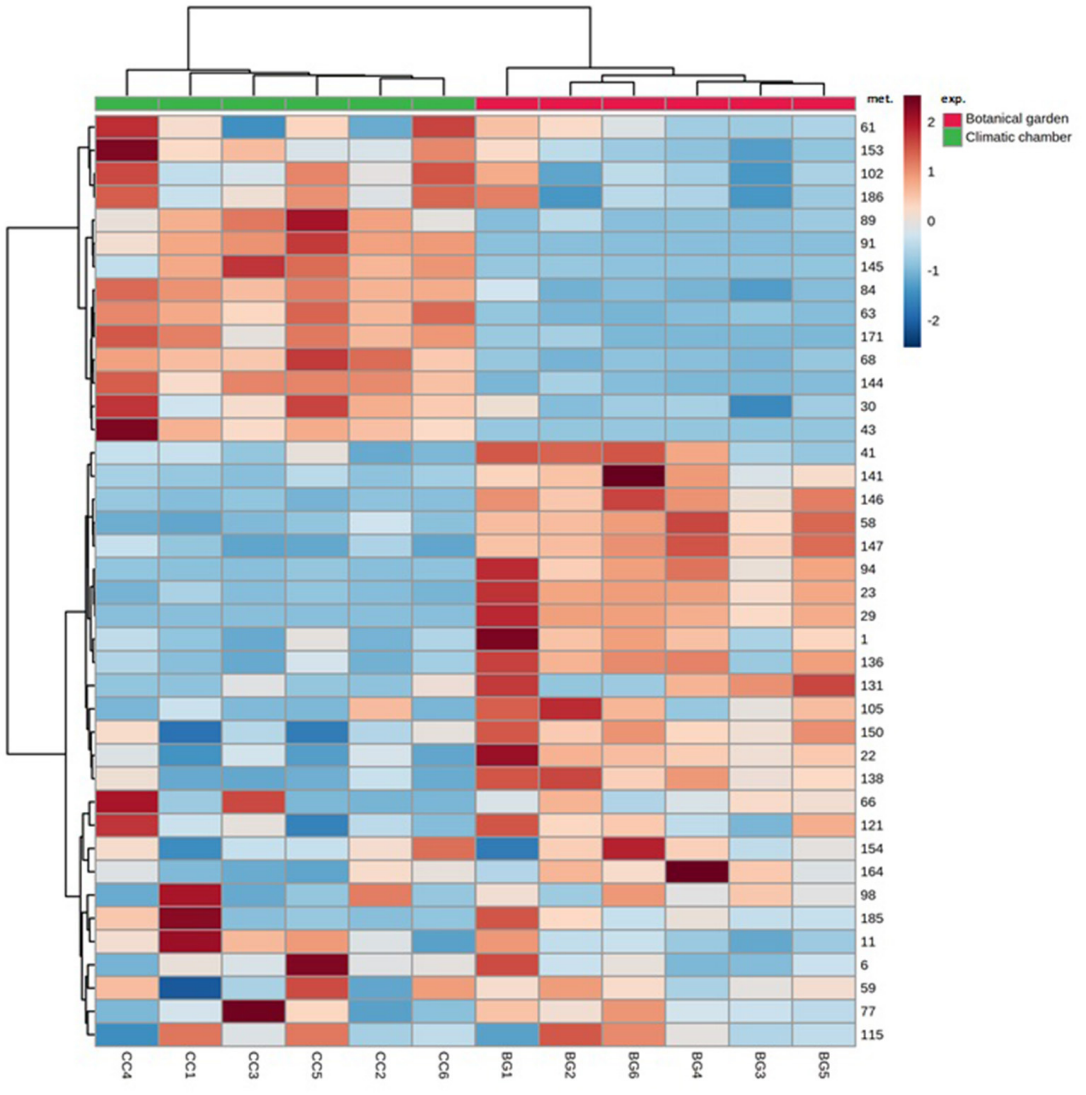
Hierarchical clustering heat maps of differentially abundant polyphenols between BG and CC treatments. For each treatment, six replicates (1–6) were performed. Significant differences were found *via* Student's *t*-test. Red: botanical garden, green: climatic chamber. The numbers in the heat maps correspond to the following metabolites (mets): (1) gallic acid; (6) trans-2-hydroxycinnamic acid; (11) 3,4,5-irimethoxycinnamic acid; (22) quinic acid; (23) 1,3-dicaffeoylquinic acid; (29) 4,5-di-O-caffeoylquinic acid methyl ester; (30) 4,5-dicaffeoylquinic acid; (41) 6,7-dihydroxycoumarin-4-acetic acid; (43) 7-diethylamino-3-formylcoumarin; (58) isoliquiritin; (59) 2-hydroxy-3-methoxychalcone; (61) 2′,4′-dihydroxy-3′-(2-hydroxybenzyl)-6′-methoxychalcone; (63) 4′,6′-dimethoxy-2′-hydroxy-4-methylchalcone; (66) 6″-O-acetylphloridzin; (68) 2′,6′-dihydroxy 4,4′-dimethoxydihydrochalcone; (77) 3,5,7,3′,4′-pentahydroxyflavanone; (84) flavanone diacetylhydrazone; (89) 4′,5,7-trihydroxy-6,8-diprenylisoflavone; (91) 6″-O-acetyldaidzin; (94) 6″-O-malonylgenistin; (98) apigenin; (102) 3,8″-biapigenin; (105) luteolin 7-methyl ether; (115) 3′,4′,5,7-tetrahydroxy-6,8-dimethoxyflavone; (121) isoorientin; (131) 6″-O-malonylisoquercitrin; (136) kaempferol 3-O-rutinoside; (138) kaempferol 3-sophoroside 7-glucoside; (141) myricetin; (144) quercitrin; (145) rutin; (146) apigeninidin-5-O-glucoside; (147) luteolinidin-5-O-glucoside; (150) (–)-catechingallate; (153) catechin 7-arabinofuranoside; (154) procyanidin A1; (164) quercetagetin-7-O-glucoside; (171) matairesinoside; (185) 2,5-dimethoxycinnamic acid; (186) miquelianin.

We sorted the annotated compounds in the following classes: phenolic acids (29 compounds sorted into three sub-classes, hydroxybenzoic, hydroxycinnamic, and quinic acids and derivatives), flavonoids (84 metabolites among antocyanins/anthocyanidins, chalcones, and derivatives, dihydrochalcones, flavanones, isoflavones, flavones, flavonols, 3-deoxyanthocyanidins, flavan-3-ols, Pas, and curcuminoids), hydrolyzable tannins (6), coumarins (15), lignans (8), and stilbenoids (4). Quinic acids and derivatives were the dominant subclass for about 98% of the total phenolic acids, which was the far most abundant class, regardless of the treatment, whereas the subclass of hydroxycinnamic acids and derivatives comprised the most significant number of phenolic acids (# 20). To investigate which phenolic subclasses and metabolites were mainly responsive to the cold stress and whether the exposure to cold could induce positive or negative modifications on such compounds, Student's *t*-test (*t*-test) for all annotated polyphenols was calculated. The results relative to the statistical analysis of subclasses are given in Figure 2, whereas those relative to the 40 metabolites that differed between treatments are given as a heat map in Figure 3. Overall, two subclasses differed significantly between the treatments: the 3-deoxyanthocyanidins and the hydroxybenzoic acids. For the former subclass, both compounds present in this category (apigeninidin 5-*O*-glucoside and luteolinidin 5-*O*-glucoside) were upregulated in the BG samples; for the latter, the unique metabolite in this group (gallic acid) was present in only in the BG samples. Within all the remaining subclasses, the differences between treatments turned out to be not statistically significant, because the presence of either slightly up- or downregulated compounds or the concomitant presence of both up- and downregulated compounds in each of them. That was the case for coumarins, in which compounds either upregulated (6,7-dihydroxycoumarin-4-acetic acid) or totally repressed (7-diethylamino-3-formylcoumarin), were found as well as for several flavonoids, such as isoflavones (with 6″-*O*-malonylgenistin upregulated and 4′,5,7-trihydroxy-6,8-diprenylisoflavone downregulated) and flavonols (with kaempferol 3-sophoriside 7-glucoside and myquelianin upregulated kaempferol 3-*O*-rutinoside and myricetin *de novo* synthesized, and quercitrin and rutin downregulated in BG). Finally, it is worthy of noting that among the upregulated compounds in BG samples, there were chalcones (isoliquiritin, 2-hydroxy-3-methoxychalcone, and 2′,4′-dihydroxy-3′-(2-hydroxybenzyl)-6′-methoxychalcone), flavanones (3,5,7,3′,4′-pentahydroxyflavanone and diacetylhydrazone), flavones (3,8″-biapigenin, isoorientin, and luteolin 7-methyl ether), and both flavan-3-ols [(–)-catechingallate and catechin 7-arabinofuranoside] and their oligomers (procyanidin A1) (Supplementary Table S3).

### Induction of *A. filiculoides* Pigmentation Under Controlled Conditions

By reasoning that cold stress coupled to moderate light intensity might represent major drivers of pigmentation in *Azolla, A. filiculoides* was grown at 5°C and 700 PPFD in a growth chamber, and the changes in the metabolic profiles were monitored over time by spectrophotometric and LC/HRMS analyses. Twenty days of the conditions above were sufficient to induce a severe red pigmentation, which, according to spectrophotometric analysis, was primarily due to the accumulation of phlobaphenes, 3-deoxyanthocyanidins (apigeninidin and luetolininidin 5-*O*-glucoside), and, to a far less extent, anthocyanidins (Table 2, Supplementary Figure S6). The same analysis revealed that 20 days of cold stress treatment doubled flavonol content and quadrupled soluble PAs, while insoluble PAs and phenolic acids remained unchanged and decreased, respectively.

**Table 2:** Polyphenols signature detected by spectrophotometric analyses in *A. filiculoides* challenged with 5°C and moderate light intensity (700 PPFD) for 20 days.

	**Anthocyanidins (nmol/gFW)**	**Phlobaphenes (Abs_**510**_/gFW)**	**Flavonols (nmol/gFW)**	**Phenolicacids (nmol/gFW)**	**Apigeninidin (nmol/gFW)**	**Luteolinidin-5-*O* glucoside (Abs_**496**_/gFW)**	**Soluble proanthocyanidins (mg catechin/gFW)**	**Insoluble proanthocyanidins (nmol/gFW)**
0 d	37.03 ± 15.85 a	2.78 ± 0.13 a	911.58 ± 71.27 a	2883.30 ± 148.29 a	63.53 ± 22.56 a	1.31 ± 0.48 a	616.76 ± 91.19 a	435.85 ± 55.04 a
1 d	34.52 ± 6.72 a	2.82 ± 0.54 a	736.92 ± 67.38 a	1633.20 ± 330.12 ab	29.45 ± 5.36 a	0.61 ± 0.08 a	604.64 ± 62.20 a	436.41 ± 58.01 a
2 d	39.06 ± 9.38 a	3.10 ± 0.65 a	677.17 ± 10.19 a	1387.52 ± 169.42 b	37.90 ± 6.48 a	0.70 ± 0.12 a	501.07 ± 57.19 a	368.26 ± 41.47 a
3 d	58.70 ± 11.71 a	4.12 ± 0.70 a	800.52 ± 73.21 a	1537.85 ± 169.47 ab	111.39 ± 26.82 a	2.14 ± 0.52 a	703.65 ± 65.14 a	357.12 ± 26.68 a
20 d	246.22 ± 23.23 b	22.36 ± 5.52 b	1739.51 ± 134.53 b	1368.71 ± 209.21 b	627.72 ± 65.99 b	13.34 ± 1.20 b	2442.47 ± 132.88 b	514.96 ± 76.84 a

*Results are means ± SE of six biological replicates. Significant differences determined by analysis of variance (ANOVA) followed by Tukey's multiple comparison test (P < 0.05 for both) are indicated by different letters*.

Then, the metabolic profile was assessed by LC/HRMS, which detected 185 metabolites vs. the 146 compounds found in the first comparison (Supplementary Table S4). Notably, among these 36 additional compounds, the vast majority (32/36) was generally barely detectable at different time points (Supplementary Table S5).

**Figure 4:**
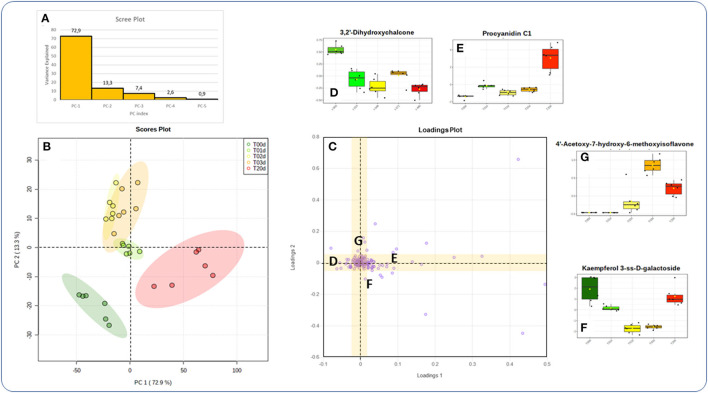
Principal Component Analysis of the data collected in the time-course experiment (700 PPFD and 5°C). **(A)** Scree plot; **(B)** score plot; **(C)** loadings plot. **(D–G)** Boxplots of metabolites representative of the change in abundance over time (D: decrease; E: increase; F: decrease, and increase; G: increase and decrease). More details in the text.

PCA of LC/HRMS data showed that the first and second components explained 73% and 13% of the overall variance, respectively (Figure 4A). The correspondent Loading Plot (Figure 4B), evidenced how the first component separated the biological replicates of the initial time point from those of the final one, while the second component those from the intermediate times from the two extreme ones.

**Figure 5:**
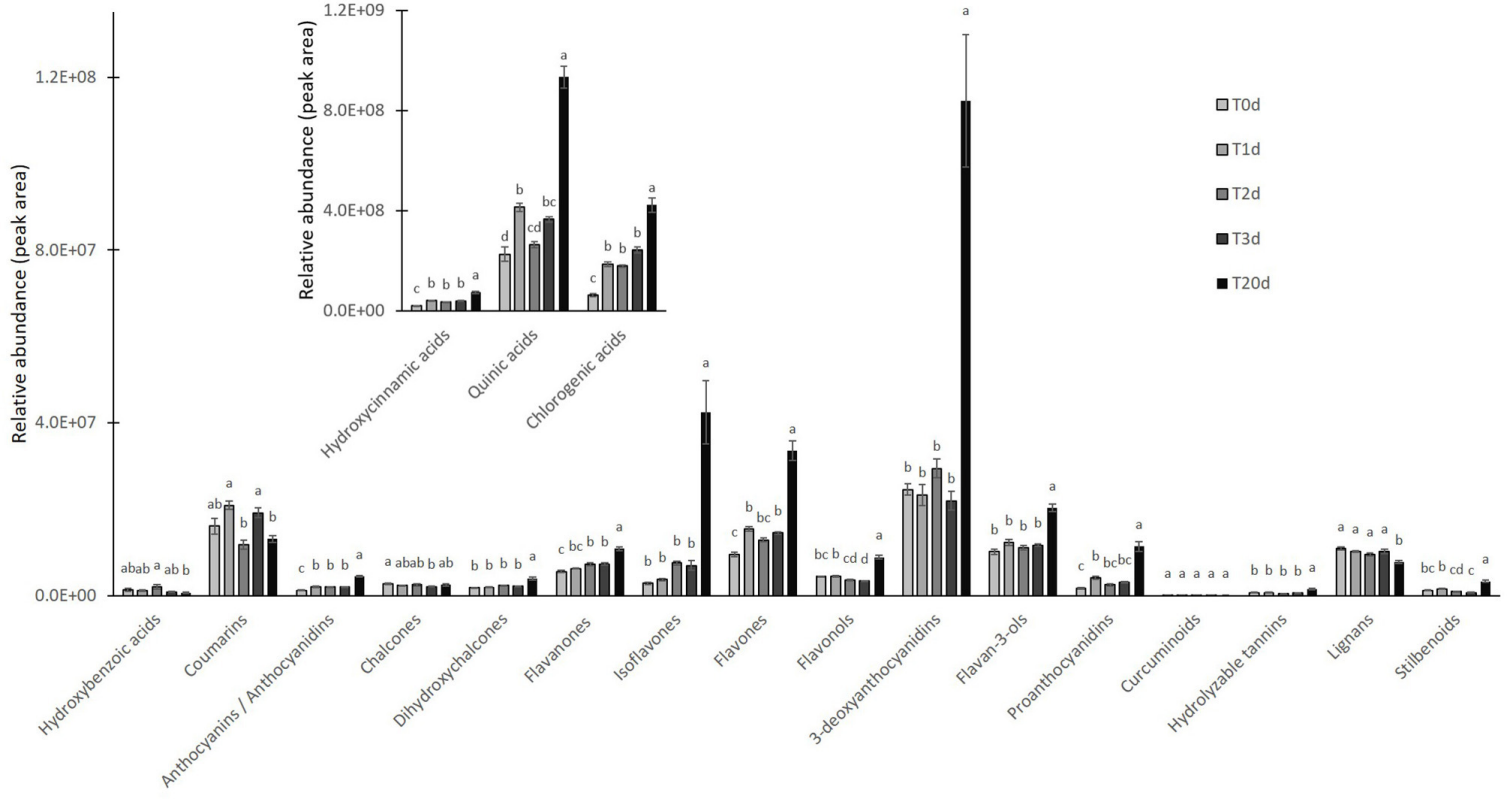
Relative abundance of different subclasses of polyphenols detected by LC/HRMS analysis in *A. filiculoides* challenged with 5°C and moderate light intensity (700 PPFD) for 20 days. Quinic acid, flavanones, and flavones were reported in a different framed histogram with higher y-axis scale for better visualization. Data are mean ± SE of six biological replicates. Different letters corresponded to statistically significance differences according to two-way ANOVA followed by Tukey's multiple comparison test (*P* < 0.001).

**Figure 6:**
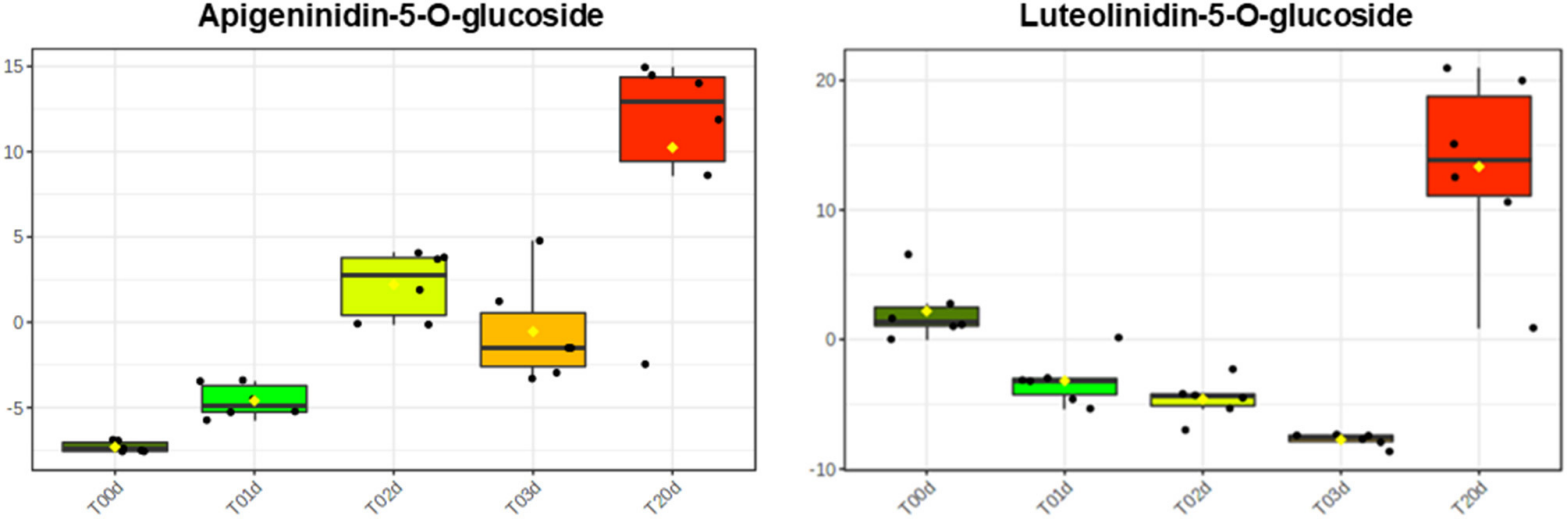
Different levels of the 3 deoxyanthoacynidins apigeninidin 5-*O*-glucoside and luetolinidin 5-*O*- glucoside in *A. filiculoides* challenged with 5°C and moderate light intensity for 20 days.

As far as the early branches of the phenylpropanoid pathway are concerned, the LC/HRMS revealed that chlorogenic acid and its derivatives increased as much as hydroxycinnamic and quinic acids at T1d to peak at T20d. Conversely, coumarins and lignans did not show a clear accumulation pattern, whereas stilbenoids and dihydrochalcones remained relatively stable until T3d to peak at T20d (Figure 5). The accumulation of different flavonoids across the treatment turned out to be quite subclass-specific. Flavanones increased over time, with those based on naringenin being the most abundant regardless of the time point considered, those on liquititigenin increasing at T20d, when those based on eriodictyol also reached their highest levels (Supplementary Table S4). The accumulation pattern of flavonols was more complex and resembled that of kaempferols, the most abundant compounds of this class. The maximum accumulation of the pigments was reached at T20d, with the less abundant anthocyanins, made up of pelargonidins, and PAs starting to increase since T1d (Figure 5). Conversely, 3-deoxyanthocyanidins, which were the most representative subclasses among the flavonoids, incremented significantly only at T20d, with the two compounds of this subclass, apigeninidin5-*O*-glucoside and luetolininidin 5-*O*-glucoside, showing a different and almost complementary trend with each other between T0d and T20d (Figure 6).

**Figure 7:**
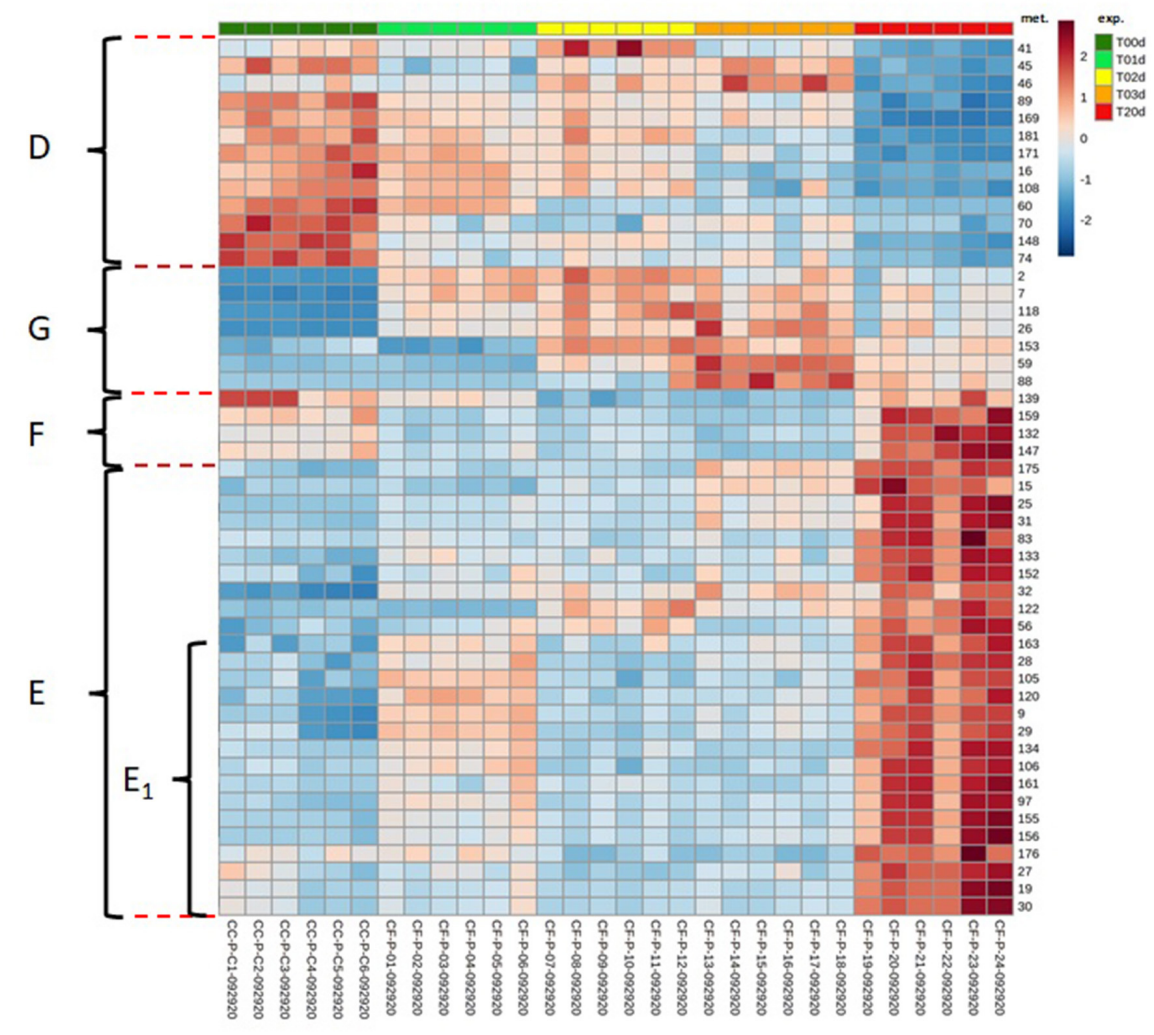
Hierarchical clustering heat maps of the data collected in the time-course experiment performed using the top 50 features from the ANOVA. The figure shows four clusters representing the main temporal trends of the metabolites. D: decrease over time, E: increase over time, F decrease and increase, G: increase and decrease. A subset of metabolites in cluster E shows a more complex trend. The numbers in the heat Maps correspond to the following metabolites (mets): (2) p-coumaric acid; (7) methyl 4-hydroxycinnamate; (9) 3-hydroxy-4-methoxycinnamic acid; (15) 1-O-cinnamoylglucose; (16) 1,6-bis-O-(4-hydroxycinnamoyl)glucose; (19) cynarin; (25) 3-O-feruloylquinic acid; (26) 3-O-p-coumaroylquinic acid; (27) 3,5-dicaffeoylquinic acid; (28) 4-O-caffeoylquinic acid; (29) 4,5-di-O-caffeoylquinic acid methyl ester; (30) 4,5-dicaffeoylquinic acid; (31) chlorogenic acid; (32) chlorogenic acid, methyl ester; (41) 6,7-dihydroxycoumarin-4-acetic acid; (45) 7-hydroxy-3-phenylcoumarin; (46) 7-hydroxy-4-methyl-3-phenylcoumarin; (56) pelargonidin cation; (59) 2-hydroxy-3-methoxychalcone; (60) 2′-hydroxy-3,4,4′,5-tetramethoxychalcone; (70) 3,2′-dihydroxychalcone; (74) liquiritin; (83) flavanone 7-O-.beta.-D-glucoside; (88) 4′-acetoxy-7-hydroxy-6-methoxyisoflavone; (89) 4′,5,7-trihydroxy-6,8-diprenylisoflavone; (97) apiin; (105) luteolin 7-methyl ether; (106) luteolin-7,3′-di-O-glucoside; (108) 7-O-benzylluteolin; (118) 4′,5,7-trihydroxy 3,3′,6,8-tetramethoxyflavone; (120) isoginkgetin; (122) licoflavone A; (132) afzelin; (133) hyperin; (134) isoquercitin; (139) kaempferol 3-ss-D-galactoside; (147) luteolinidin-5-O glucoside cation; (148) (+)-catechin; (152) (–)-gallocatechin 3-gallate; (153) catechin 7-arabinofuranoside; (155) procyanidin B2; (156) procyanidin C1; (159) 1,2,3,4,6-pentagalloyl.beta.-D-glucose; (161) 1,3,6-tri-O-galloyl-.beta.-D-glucose; (163) 6-O-p-coumaroyl-1,2-di-O-galloyl-.beta.-D-glucopyranose; (169) enterodiol; (171) matairesinoside; (175) silydianin; (176) deoxyrhapontin; (181) di-p-coumaroylputrescine.

When single metabolites were considered, the hierarchical clustering heat maps created using the top 50 features from ANOVA showed that they could be sorted into four main temporal clusters, named from D to G. Clusters D and E grouped metabolites that decreased and increased, respectively, from T0d to T20d, cluster F grouped metabolites that decreased at intermediate time points to increase at T20d, and cluster G grouped metabolites that increased at intermediate time points to decrease at T20d (Figure 7). The numerically most abundant cluster was E, within which some metabolites showed a more complex pattern (E_1_) in that they decreased between T1d and T2d. Metabolites belonging to phenolic acids, flavones, flavonols, and PAs were mainly present within clusters E and F, although other metabolites of these classes were also present in the D and G clusters. The Loading plots tool of the Metaboanalyst program enables the identification of metabolites that are the most responsible for driving the patterns seen in the score plot (Figure 4C). The accumulation pattern of each single metabolite traced using the tool mentioned above showed that among the metabolites arranged on the axis of the first component, in which compounds that decreased or increased from the initial to the final time point are present, were a dihydroxychalcone (3,2′-dihydroxychalcone) (Figure 4D) and the PAs B2 and C1 (Figure 4E, data not shown), respectively. Conversely, among the metabolites arranged on the axis of the second component, corresponding to metabolites that decreased or increased at intermediate times concerning both the initial and final time points were a flavonol (kaempferol 3-SS-D-galactoside) (Figure 4F), and an isoflavone (4′acetoxy-7-hydroxy-6-methoxyisoflavone) (Figure 4G), respectively.

### Selection of *A. filiculoides* Candidate Phenylpropanoid Genes for Targeted Expression Profiling

In *A. filiculoides*, as in other plant species, early key enzymes of the phenylpropanoid pathway are coded by small gene families (Piatkowski et al., 2020; Güngör et al., 2021). An exception to this rule is *CHI*, for which only a single gene was identified in the genome of this fern. To gain an overview on how the *PAL, CH4*, and *CHS* gene families, rather than single-gene members, respond to changing environmental conditions, the seven, two, and seven genes present in these families, respectively, were aligned and used as primers for qRT-PCR analysis designed on highly conserved domains for each family along with a primer pair for *CHI*. Later steps on the phenylpropanoid are coded by genes belonging to gene-rich families *Cytochrome P450* (*flavone synthase II, FNSII*; *isoflavone synthase, IFS*; *flavonoid 3*′ *hydroxylase, F3*′*H*; and *flavonoid 3*′*5*′ *hydroxylase, F3*′*5*′*H*), *2-oxoglutarate-dependent dioxygenase* (*2OGD*) (*F3H, flavone synthase I, FNSI*; *flavanol synthase, FLS;* and *leucocyanidin oxygenase/anthocyanidin synthase, LDOX*/*ANS*), and *short-chain dexydrogenase/reductase* (*SDR*) (*DFR, FNR*, and *LAR*) with candidate *A. filiculoides F3*′*H, DFR, LAR*, and *FLS/ANS* genes already reported (Piatkowski et al., 2020; Güngör et al., 2021). Following the above criteria, primer pairs were designed for the expression analysis of *DFR, F3*′*H*, and *LAR* and reported in Supplementary Table S2. Primers were also designed on two *SDR* ones, namely, Azfi_s0008.g011655 and Azfi_s0008.g011657, showing 70% of identity at nucleotide level each other and identity to the two *DFR* genes identified by Güngör et al. (2021) ranging from 51 to 61%. The former two genes formed a sister clade in the phylogenetic tree of SDR proteins with respect to the latter two genes (Supplementary Figure S7) to suggest the presence of at least two *DFR* gene families in *A. filiculoides*: *DFR1* consisting of Azfi_s0035.g025620 and Azfi_s0245.g059984, and *DFR2* with Azfi_s0008.g011655 and Azfi_s0008.g011657.

Finally, primers were designed on the following *2OGD* genes: *Azfi_s0335.g065519, Azfi_s0076.g037942, Azfi_s0039.g026462*, and *Azfi_s0076.g037826*. The first two genes were reported by Güngör et al. (2021) as *F3H*-like. If our phylogenetic analysis supported, albeit, with low boostraps, the relatedness of Azfi_s0076.g037942 to an F3H gene from Arabidopsis (NP_190692), it did not work for Azfi_s0335.g065519. This last entry conversely shared 44% identity at amino acidic level with Azfi_s0039.g026462, and both were in a clade next to that grouping FLS and some ANS from higher plants, but different from that of Azfi_s0076.g037826, an FLS/ANS-like according to Güngör et al. (2021) (data not shown).

### Targeted Gene Expression Profiles in Fronds From *A. filiculoides* Under Control and Pigment-Inducing Conditions

**Figure 8:**
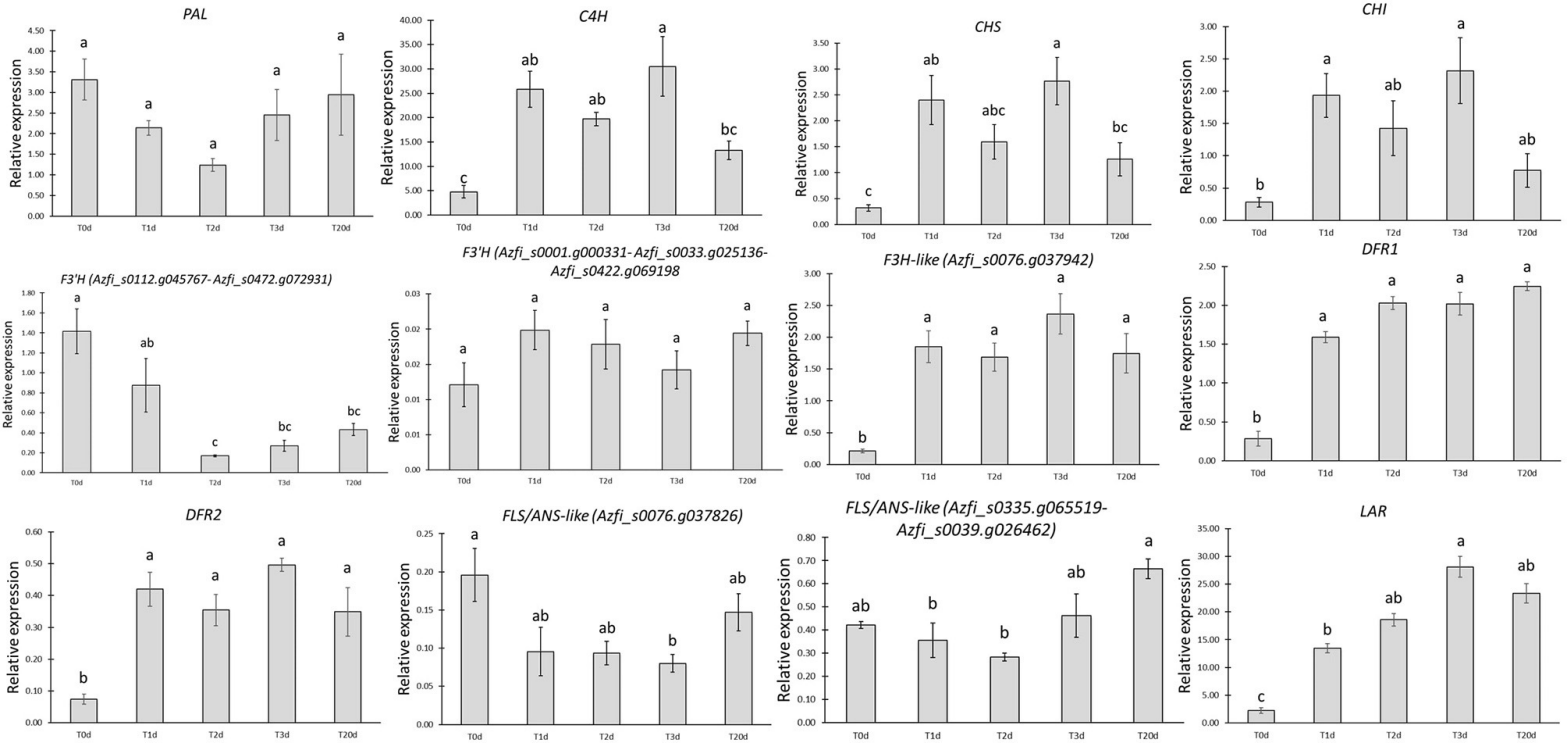
Relative expression of early and late genes/gene families in the time-course experiment. The relative expression of each gene/gene family is calculated using the (2^−Δ*Ct*^) algorithm. Significant differences determined by ANOVA followed by Tukey's multiple comparison test (*P* <0.05 for both) are indicated by different letters.

A subset of gene families or genes consisting of *PAL, CHS, CHI, DFR*, and *LAR* was initially investigated for their expression in the BG and CC samples using the *elongation factor* 1α gene as reference. This gene showed a steady expression in a previous study on *Azolla* and other plant species challenged with stress (Paolocci et al., 2005; de Vries et al., 2018). Early genes were downregulated in the BG samples, whereas the late ones were upregulated (data not shown). Then, the expression profiles of the most important key genes of the phenylpropanoid pathway were monitored in *Azolla* from the onset of the cold and medium light treatment for up to 20 days when the change in color was almost entirely achieved in all the plants (Figure 8). Interestingly, except for *PAL*, whose expression was not significantly different over the treatment, the other early gene (*CHI*) or gene families (*C4H* and *CHS)* investigated showed significant upregulation since the T1d of treatment. Indeed, they showed bimodal patterns, peaking at T1d and T3d to decrease at 20 days. Among these early genes, *C4H* displayed the highest mRNA levels, regardless of the time point considered. The steady-state levels of the putative *F3H* gene *Azfi_s0076.g037942*, the two *DFR1* genes, and *LAR* were conversely sustained and higher than the control all along the pigment-inducing treatment, with that of *LAR* peaking at 72 h. Notably, *LAR* showed highest fold change increments since T1d concerning all the other late genes investigated. The two SDR genes, *Azfi_s0008.g011655* and *Azfi_s0008.g011657*, assigned to the *DFR2* gene family also showed a significant increment since T1d of the onset of the treatment, but their steady-state levels were consistently lower than those of the *DFR1* gene family. The putative *FLS*/*ANS* genes tested remained pretty stable across the treatment, even if *Azfi_s0335.g065519* and *Azfi_s0039.g026462* declined significantly at T2d, and *Azfi_s0076.g037826* at T3d. Among the *F3*′*H*genes investigated, the combined steady-state levels of *Azfi_s0112.g045767* and *Azfi_s0472.g072931* resulted to be substantially lower than the control since T48h, whereas those of *Azfi_s0001.g000331, Azfi_s0033.g025136*, and *Azfi_s0422.g069198* were stable during the treatment, but their levels was at least one order of magnitude less than those of *Azfi_s0112.g045767* and *Azfi_s0472.g072931* (Figure 8).

## Discussion

Aside from its use as fertilizer in rice paddies, the potential use of *Azolla* spp. as a fast-growing and sustainable plant material for diverse applications such as feeding, biofuel production, and bioremediation has encountered a growing interest over the last years (Carrapico et al., 2000; Bennicelli et al., 2004; Brouwer et al., 2014; Kollah et al., 2016; Fernández, 2018). However, comprehending the array of secondary metabolites and their dynamics under changing environmental clues in fern species is still in its infancy. To fill in this gap, here, we performed spectrophotometric and LC-HRMS analyses on *A. filiculoides* harvested outdoor, when it showed red fronds, and on *A. filiculoides* grown under controlled conditions, when it showed green fronds. We also evaluated samples challenged for up to 20 days with cold stress coupled to moderate light intensity. These metabolic analyses were paralleled by the targeted qRT-PCR analysis of a group of candidate structural genes of the phenylpropanoid pathway. Here, we show that the reddish color induced by stress is due to a combination of 3-deoxyanthocyanidins, phlobaphenes, and a far less amount of anthocyanins, and that along with these pigments and the already reported PAs (Markham, 1988; Güngör et al., 2021), this fern is competent to accumulate other secondary metabolites of outstanding importance, such as chemoattractants, defense compounds, and ROS scavengers, and crucial as dietary components for humans such as dihydrochalcones, stilbenes, and isoflavones (Rivière, 2016; El Khawand et al., 2018; KríŽová et al., 2019).

### Different Involvement of Pigments in the Red Color of *A. filiculoides* Collected Outdoor

In *A. filiculoides*, seasonal fluctuation triggers changes in chlorophyll a/b ratio, levels of carotenoids and anthocyanins, and different growth rates (Kosesakal, 2014). The reddish color observed in *A. filiculoides* under high light intensity and low temperature conditions was attributed to the accumulation of anthocyanins. In contrast, Nham Tran et al. (2020) documented a more than 2-fold increase in the production of total phenols, an 18-fold increase in the production of anthocyanins, and up to 2.7 fold increase in the production of condensed tannins in *A. filiculoides* plants grown outdoor and stressed by a combination of starving and direct exposure to high light intensity (5–10 Klux daytime) vs. shaded plants grown outdoor without starvation. Changes in the pigment and phenolic accumulation of *A. filiculoides* challenged with sodium dodecyl sulfate (SDS) or metals have also been described (Sánchez-Viveros et al., 2010, 2011; Dai et al., 2012; Forni et al., 2012). By LC-MS analysis, the first glimpse of phenolic compounds that accumulated in *A. filiculoides* was achieved (Brouwer et al., 2017). By comparing the spectrophotometric profiles of phenylpropanoids of the red *Azolla* grown outdoor and upon its acclimation to the growth chamber conditions, we found higher levels of anthocyanins, flavonols, soluble and insoluble Pas, and, remarkably, of the two 3- deoxyanthocyanins, apigeninidin and luteolinidin5-*O* glucoside, and phlobaphenes in the samples harvested outdoor. Although their distribution is sporadic in ferns and bryophytes, the occurrence of 3-deoxyanthocyanins, especially luteolinidin 5-*O*-glucoside, in the *Azolla* genus has been regarded as a stable taxonomic chemical marker for this genus by Iwashina et al. (2010) being luteolinidin 5-*O* glucoside, the pigment responsible for the red color in basically all the *Azolla* spp. investigated thus far (Holst, 1977; Ishikura, 1982). The biosynthesis of 3-deoxyanthocyanins occurs in those species in which the lack or strong suppression of the *F3H* gene, which codes for the entry point enzyme for anthocyanins synthesis, promotes the conversion of flavanones into flavan-4-ols apiforol and luteoforol, likely because of the activity of a DFR-like enzyme as it occurs in sorghum (Liu et al., 2010). In turn, flavan-4-ols are converted into 3-deoxyanthocyanins, likely by the activity of an ANS-like enzyme, or polymerized to give off phlobaphenes with reddish insoluble pigment (Casas et al., 2014). Interestingly, to the best of our knowledge, this study is the first to document the accumulation of phlobaphenes in *Azolla* spp. The occurrence of PAs in *Azolla* spp. has been documented for decades (Markham, 1988), with these stress-inducible compounds thought to be the most abundant soluble phenolics in both *A. filiculoides* and *Azolla pinnata* (Nham Tran et al., 2020). On this ground, both the spectrophotometric and LC-HRMS analyses documented higher levels of PA in the BG samples. Additionally, the untargeted metabolic analysis also showed that besides the compounds already characterized in *Azolla* spp., such as p-coumaric, ferulic, and chlorogenic acids and their derivatives, fatty acids, and lignans (Brouwer et al., 2017; Qian et al., 2020), *A. filiculoides* is indeed competent to synthesize dihydrochacones, stilbenes, hydrolazable tannins, isoflavones, and curcuminoids. The abundance of these compounds is similar to that of anthocyanins under the two growing conditions tested, with delphinidin-based compounds being the most present in this subclass.

### The Time-Course Analysis Reveals That the Stress-Induced Color Transition in *A. filiculoides* Fronds Is a Visible Marker of a More Significant Number of Changes in the Phenylpropanoid Pathway

What we monitored in the BG samples collected in February could be the result of metabolic changes that occurred much earlier. Likewise, aside from the visible color transition, nothing is known on the timing of the turnover of pigments and other phenylpronoids in this fern as a result of changes in light, temperature, and other physical, chemical, and nutritional parameters, which could not be controlled at the collection site. Thus, we decided to investigate the dynamics of phenylpropanoids in *A. filiculoides* over 20 days of the controlled growing conditions under cold stress coupled to moderate light intensity. Twenty days under these conditions is sufficient to cause a severe reddish pigmentation of the fronds. As per the spectrophotometric analysis, a significant increment of the pigments phlobaphenes, 3-deoxyanthocyanidins, and soluble PA is achieved only at T20d, along with that of flavonols, whereas the levels of insoluble PAs do not change along the treatment, and that of phenolic acids is significantly lower at T2d and T20d than at T0h. However, this decrement in phenolic acids was not confirmed by the more sensitive LC-HRMS analysis. Rather, this analysis showed that phenolic acids are the far most abundant class of phenylpropanoids all over the treatment. Moreover, they responded more quickly than others to changing environmental conditions: hydoxycinnamic acids, quinic and chlorogenic acids, and their derivatives, but no hydrobenzoic acids increase since T1d. Whether *Azolla* spp. are competent to synthesize lignin is a long-lasting debate (Nierop et al., 2011), and the finding that among the *A. filiculoides* hydroxycinnamic acid derivatives that peak at T20d there is sinapyl alcohol supports the idea that under specific conditions, *A. filiculoides* is competent to synthesize at least one of three essential monolignols of plant lignin (Boerjan et al., 2003). The plasticity of the early step of *A. filiculoides* phenylpropanoid pathway for different treatments and likely plant stage might explain why chlorogenic acids and their derivatives have not been found in all the conditions tested. The other products of the early branches of the phenylpropanoid pathway show contrasting accumulation patterns, and only hydrolyzable tannins and stilbenoids reach their maximum at the end of the color-inducing treatment. Aside from their roles as defense compounds against microbial pathogens, stilbenes (Dubrovina and Kiselev, 2017) or herbivores, hydrolyzable tannins (Haslam, 1988), the accumulation of these compounds, as well as that of hydroxycinnamic and chlorogenic acids, seems to arise in virtue of their antioxidant properties (Agati and Tattini, 2010). The increase in ROS levels, followed by the activation of enzymatic and non-enzymatic defense ROS scavenging systems, is a conserved response to cold stress across the plant kingdom (Huang et al., 2019). In this context, activating the late branches of the phenylpropanoid pathway, those of flavonoids, is an expected result.

### *A. filiculoides* As Biofactory of a Vast Array of Stress Avoidance Flavonoids

Flavonoids can be classified as open- or closed-chain flavonoids (Ibdah et al., 2018). Different from the former group of flavonoids, the open-chain ones, represented by dihydochalcones, do not rely on the activity of CHS and CHI enzymes for their biosynthesis. Both open- and closed-chain flavonoids had an increment at T20d, although with different trends. If dihydrochalcones, isoflavones, deoxyanthocyanidins, and flavan-3-ols remain stable up to T3d, anthocyanins, flavanones, flavones, and PAs will show a step-wise increment, with generally higher levels between T1d and/or T3d than T0d. In contrast, a more complex pattern is demonstrated by flavolons, which decrease after 2 days of treatment.

That flavonoids peak at T20d is in keeping with their role as one of the primary non-enzymatic antioxidants (Gechev et al., 2006). Likewise, although the photoprotective role of flavonoids is still controversial (Agati et al., 2020), the accumulation of flavonoid pigments in the epidermal cells of stressed *A.filiculoides* fronds could be seen as a “biochemical adjustment” to attenuate the flux of damaging solar wavelengths to leaf targets made sensitive by the excess of cold-induced ROS. Therefore, a study is in progress to disentangle the role and contribution of cold and light stressors in shaping the metabolome of this fern. It also remains to be verified whether and to what extent the changes in the profiles of important plant microbial signaling molecules, such as isoflavones and deoxyanthocyanins (Cohen et al., 2002b), induced by our stressful condition might affect the composition and nitrogen fixation efficiency of the *Azolla-Trichormus* superorganism. Given that cyanobacteria are known to synthesize phenolic compounds (Blagojevic et al., 2018), future studies will be also needed to assess the contribution of *Trichormus* symbiont to the overall accumulation of phenylpropanoids isolated from *Azolla* fronds under different environmental conditions.

Our investigation shows how rich in branches and complex is the flavonoid pathway in this fern. Precursors of closed-chain flavonoids are flavanones. Our time-course analysis reveals that not only flavanones with two (eriodictyol), one (naringenin), and no (flavanone) hydroxyl groups in their B-ring but also liquiritigenin are accumulated among this class of flavonoids. Still better, the difference in trends and levels along with the cold treatment emerged within them. From the evidence that: (a) the levels of naringenin-based flavanones are higher than those of the others at any time point; (b) among flavonols and anthocyanins those with one hydroxyl group in their B-ring, kaempferols and pelargonidins, respectively, are the most abundant; (c) luetolins, genisteins, and apigenidin 5-*O* glucoside are produced from naringin *via* FLS, ISF, and DFR, respectively, let us argue that the metabolic flux through naringenin is predominant over those stemming from other flavanones. Additionally, as the levels of luteolinidin 5-*O* glucoside produced from eriodictyol *via* FLS is higher than that of apigenidin 5-*O* glucoside, we are tempted to speculate that eriodyctiol serves essentially as the substrate for the production of the former deoxyanthocyanins and phlobaphenes *via* luteoforol. As such, the levels of luteolinidin 5-*O* glucoside phlobaphenes and eriodictyols all peak at T20d. Along with the reasoning above, liquiritigenin could mainly serve as the substrate for the production of isoflavonesdiadzins and formononetin. However, the hydroxylation of intermediate metabolites by means of F3′H and F3′5′H enzymatic activities should occur at later stages than that of flavanones and under specific conditions. This could explain why: (a) no flavan-3-ols with a single (afzelechins) but those with two (catechins) or three (gallocatechins) hydroxyl groups in the B ring have been recorded; (b) the anthocyanins with one (pelargonidins) and two (delphinidins) hydroxyl groups in this ring are the most abundant in artificially and naturally induced reddish fronds, respectively. In keeping with this, we note that the *F3*′*H* genes are expressed, with a couple of them (Azfi_s0112.g045767 and Azfi_s0472.g072931) that, after dropping at T2d, slightly increase at T20d, and that the *F3*′*H5*′*H* genes remain to be identified in the *A. filiculoides* genome (Güngör et al., 2021).

### Targeted Gene Expression Analysis Reveals a Different Timing Between Early and Late *A. filiculoides* Genes of the Phenylpropanoid Pathway in Response to Environmental Stress

The targeted qRT-PCR analysis largely corroborates what has been shown by the untargeted metabolomics carried out over the 20 days of stress treatment. Except for *PAL*, all the other early gene families of the phenylpropanoid pathway tested (*CHS, CHI*, and *C4H*) clearly show an upregulation since the beginning of the treatment, and a bimodal patterns with general peaks at T1d and T3d followed by a decrease at T2d and T20d, when their steady-state levels were not statistically dissimilar to that of T0h. Of note, among these early gene families, the two *C4H* genes tested show the highest steady-state mRNA levels at any stage tested. The upregulation of these genes likely serves to sustain the parallel biosynthesis of both closed- and open-chain flavonoids. The approach we pursued focused on assessing the expression profile of gene families profile rather than that of each single gene member. Therefore, we cannot rule out the hypothesis that single members within each family might respond differently from others to a given treatment, as already shown, for instance, for members of the *CHS* and *DFR* gene families in *Lotus corniculatus* plants challenged with different light regimes (Paolocci et al., 2005).

The analysis of the two *DFR* genes and the functionally tested *LAR* gene identified by Güngör et al. (2021) shows how late genes of the flavonoid pathway remain upregulated from T1d to T20d and suggest that early and late gene families of this route undergo different genetic and environmental control as it also occurs in higher plants (Hichri et al., 2011; Petroni and Tonelli, 2011). The presence of the two *DFR* genes, namely *Azfi_s0008.g011655* and *Azfi_s0008.g011657*, with a similar expression pattern but a steady-state level lower than those already characterized prompts us to argue on the presence of at least two *DFR* gene families in the *Azolla* genome, each with a specific pathway commitment. Very recently, de Vries et al. (2021) have also shown that the four DFR proteins present in *A. filiculoides* cluster into two different branches. Besides, they reported that these proteins from *Azolla*, along with two from *Salvinia cucullata*, form a fern-specific DFR/DFR-like clade. DFR enzymes not only play a critical role in the biosynthesis of leucoanthocyanidins from dihydroflavonols and, hence, of anthocyanidins, flavan-3-ols, and PAs downstream, but they are also thought to be involved in the reduction of the flavanones naringenin and eriodictiol into apiforol and luteoforol, respectively, which are the building blocks of phlobaphenes (Liu et al., 2010). Therefore, this study paves the way for functional analysis to test whether *DFR* genes within and between these two gene families code for enzymes with different substrate specificity. The high expression levels of *LAR* throughout the stress treatment is consistent with the increment of both flavan-3-ols and PAs as testified by spectrophotometric, LC/HRMS analysis, and histochemical staining. In fact, LAR reduces leucoanthocyanidins into the flavan-3-ol catechin (Tanner et al., 2003; Paolocci et al., 2007) and by converting 4β-(S-cysteinyl)-epicatechin (Cys-EC), an epicatechin-type extension unit for non-enzymatic PA polymerization, into an epicatechin starter unit, it plays a critical role in controlling the degree of PA polymerization (Liu et al., 2016; Yu et al., 2019).

Apart from the genes mentioned above, for which functional annotation is well-supported by phylogenetic analysis as per previous studies, the role of enzymes coded by the other genes investigated in this study remains to be elucidated. In this concern, whereas (Piatkowski et al., 2020) concluded that fern genomes lack any orthologs of *F3H*, Güngör et al. (2021) identified the *Azfi_s0335.g065519* and *Azfi_s0076.g037942* entries as two putative *A. filiculoides F3H* genes. Our phylogenetic tree poses these two entries into two different branches of the 2OGD tree, and the gene-specific qRT-PCR data show that *Azfi_s0076.g037942* displays an expression pattern resembling those of the *DFR* and *LAR* genes, and that *Azfi_s0335.g065519* is upregulated only at T20d. Overall, conclusive evidence on the presence and function of *F3H* genes in *Azolla* spp. cannot be drawn. However, the presence of metabolites derived from the activity of the enzymes coded by these gene(s) and the expression pattern of *Azfi_s0076.g037942*, let us infer that a gene functioning as *F3H* is present in *A. filiculoides*, and that the gene is *Azfi_s0076.g037942*.

The present study's findings open to future research of both fundamental and pragmatic relevance. The evidence that *Azolla* is competent to synthesize a vast array of phenylpropanoids, some of which occur with a limited and heterogeneous distribution in the plant kingdom, set the stage for future high-throughput transcriptomics analyses to identify both structural and regulatory genes controlling the different branches of the phenypropanoid pathway. The amenability to grow under controlled and stressful conditions and the fact that its genome has been sequenced make *A. filiculoides* a model to gain evidence on the genetic control of secondary metabolites in lower plants and trace its evolution/conservation across plant species. From a pragmatic point of view, the high growth rate and the possibility to elicit, upon application of moderate stresses, the synthesis of metabolites central to human health and food/beverage processing, such as dihydrochacones, stilbenes, isoflavones, deoxyanthocianins, phlobaphenes, and PAs, lay the groundwork on which to build future studies aiming at assessing the potential role of this fern as a sustainable source of beneficial metabolites for humans.

## Data Availability Statement

The datasets presented in this study can be found in online repositories. The names of the repository/repositories and accession number(s) can be found in the article/Supplementary Materials. The ITS sequence of the *Azolla* specimen analysed in this study is deposited in GenBank under the accession number MW872256. All raw data from the LC/MS analyses supporting the conclusions of this article will be made available by the authors, without undue reservation.

## Author Contributions

FP and SP conceived the research, drafted, and wrote the manuscript. RP, AC, and SC performed metabolic and statistical analyses. MC and LR carried out morphological analysis. FP and AC performed molecular analysis. AC and SC carried out the experimental setup. FP, SP, and RP performed interpretation of the data. All the authors read and improved the manuscript.

## Funding

This study was supported by the national project MIUR–PRIN 2017 (Grant No: 2017N5LBZK_004).

## Conflict of Interest

The authors declare that the research was conducted in the absence of any commercial or financial relationships that could be construed as a potential conflict of interest.

## Publisher's Note

All claims expressed in this article are solely those of the authors and do not necessarily represent those of their affiliated organizations, or those of the publisher, the editors and the reviewers. Any product that may be evaluated in this article, or claim that may be made by its manufacturer, is not guaranteed or endorsed by the publisher.

## Supplementary Material

Supplementary Figure S1 Maximum, minimum, and mean temperatures recorded during the period October 2019–February 2020 provided by the Hydrographic Service of the Regione Umbria **(A)**. Data of global solar radiation, measured as W/m^2^ and PPFD (μmoles m^−2^ s^−1^), provided by the ARPA Umbria (http://www.arpa.umbria.it) **(B)**.

Supplementary Figure S2 Color of *Azolla* sampled in the botanical garden **(A)** vs. that grown in the climatic chamber **(C)**. Image of the upper lobes of *Azolla* grown in the botanical garden **(B)**.

Supplementary Figure S3 Change in the color of *Azolla* fronds under **(A)** high light and **(B)** shading conditions.

Supplementary Figure S4 Alignment of nuclear ribosomal DNA (rDNA) internal transcribed spacer (ITS) from *Azolla* spp. MW872256 (*A. filiculoides* collected from BG-PG); *A. filiculoides* (JX297314); *A. rubra* (DQ066492); *A. microphylla* (DQ066487); *A. mexicana* (DQ066477); *A. caroliniana* (DQ066473); *A. pinnata* (DQ066490); *A. nilotica* (DQ066470).

Supplementary Figure S5 Principal component analysis (PCA) score plot of the 146 polyphenols annotated from the six replicated samples for each growing condition tested in the CC vs. BG comparison. Total quantitative variances were clustered to reveal differences and relative similarities of different samples. 95% confidence regions (Hotelling's T2 eclipse) are displayed for each class.

Supplementary Figure S6 Change in the color of *Azolla* fronds during the cold stress treatment under controlled conditions; on days **(A)** 0, **(B)** 1, **(C)** 2, **(D)** 3, and after **(E)** 20 days of treatment.

Supplementary Figure S7 Neighbor-joining tree of dihydroflavonol 4-reductase (DFR) proteins. The four *DFR* coding genes considered in this study are marked. The tree was built using 1,000 bootstraps.

Supplementary Table S1 Data annotation of metabolites.

Supplementary Table S2 List of the genes investigated by qRT-PCR analysis and sequence of the primers employed.

Supplementary Table S3 Metabolic data relative to the CC and BG samples.

Supplementary Table S4 Metabolic data of *A. filiculoides* in the time-course experiment under controlled stress conditions.

Supplementary Table S5 Phenylpropanoids present in the time-course experiment but absent in the CC vs. BG comparison.
