# Supplementary figures and images for: Light and Temperature Shape the Phenylpropanoid Profile of Azolla filiculoides Fronds
Source: Front Plant Sci. 2021 Oct 21;12:727667. doi: 10.3389/fpls.2021.727667 (PMC8567065; doi:10.3389/fpls.2021.727667)

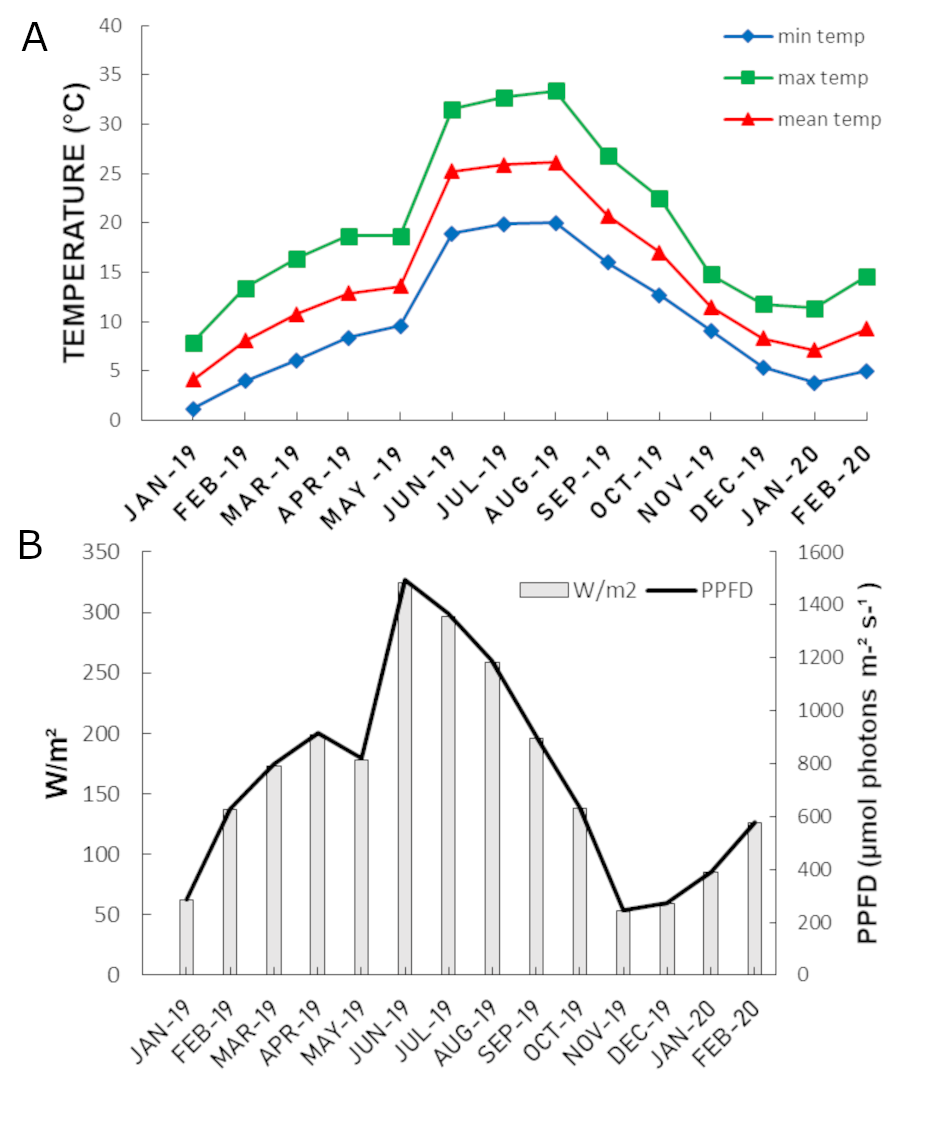

Supplement: Supplementary Figure S1 — Maximum, minimum, and mean temperatures recorded during the period October 2019–February 2020 provided by the Hydrographic Service of the Regione Umbria (A). Data of global solar radiation, measured as W/m2 and PPFD (μmoles m−2 s−1), provided by the ARPA Umbria (http://www.arpa.umbria.it) (B). [file Image_1.TIF]

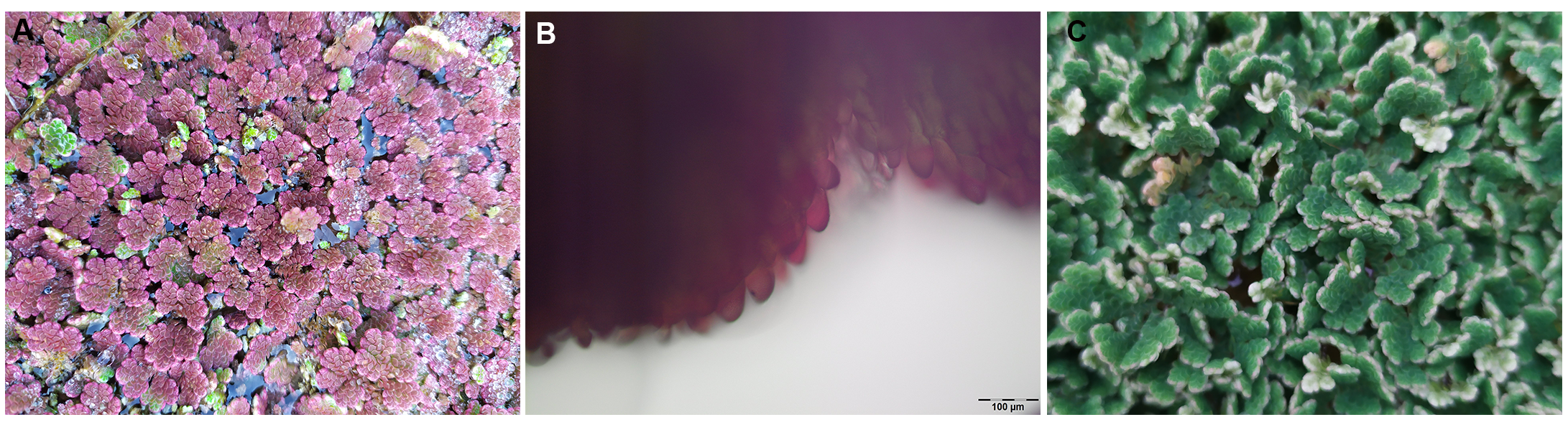

Supplement: Supplementary Figure S2 — Color of Azolla sampled in the botanical garden (A) vs. that grown in the climatic chamber (C). Image of the upper lobes of Azolla grown in the botanical garden (B). [file Image_2.TIF]

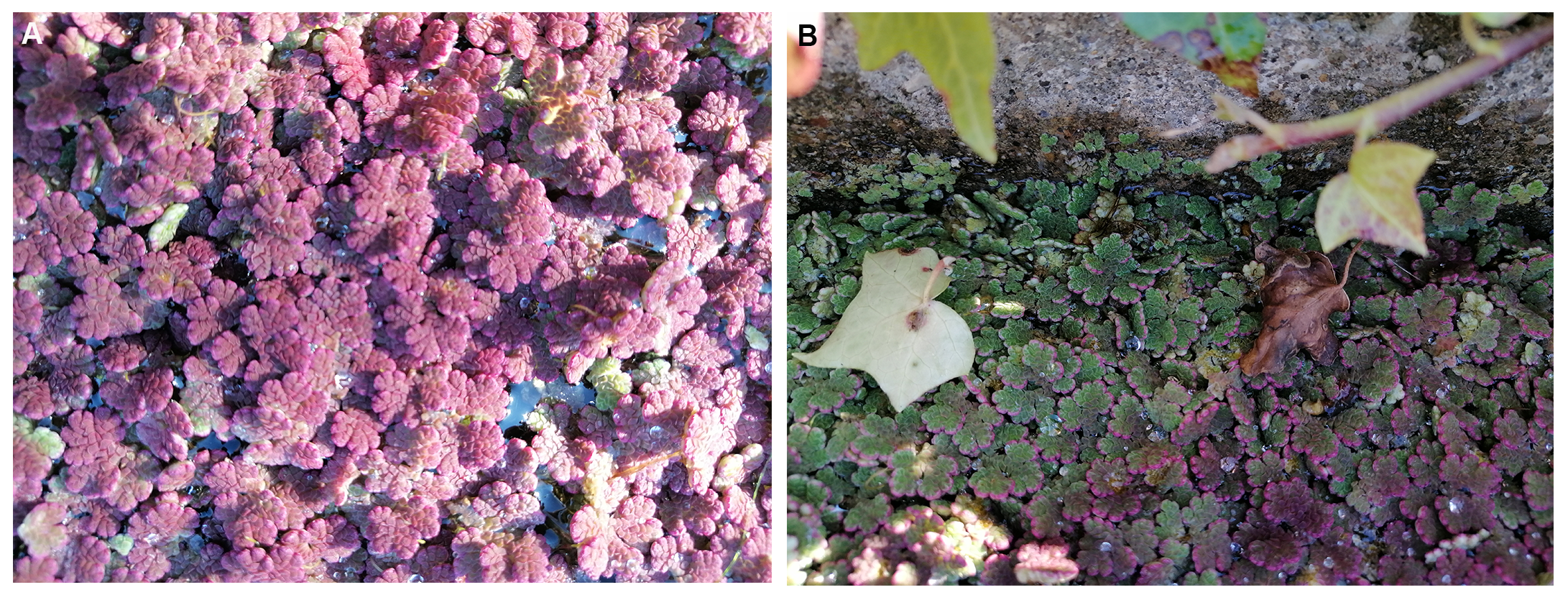

Supplement: Supplementary Figure S3 — Change in the color of Azolla fronds under (A) high light and (B) shading conditions. [file Image_3.TIF]

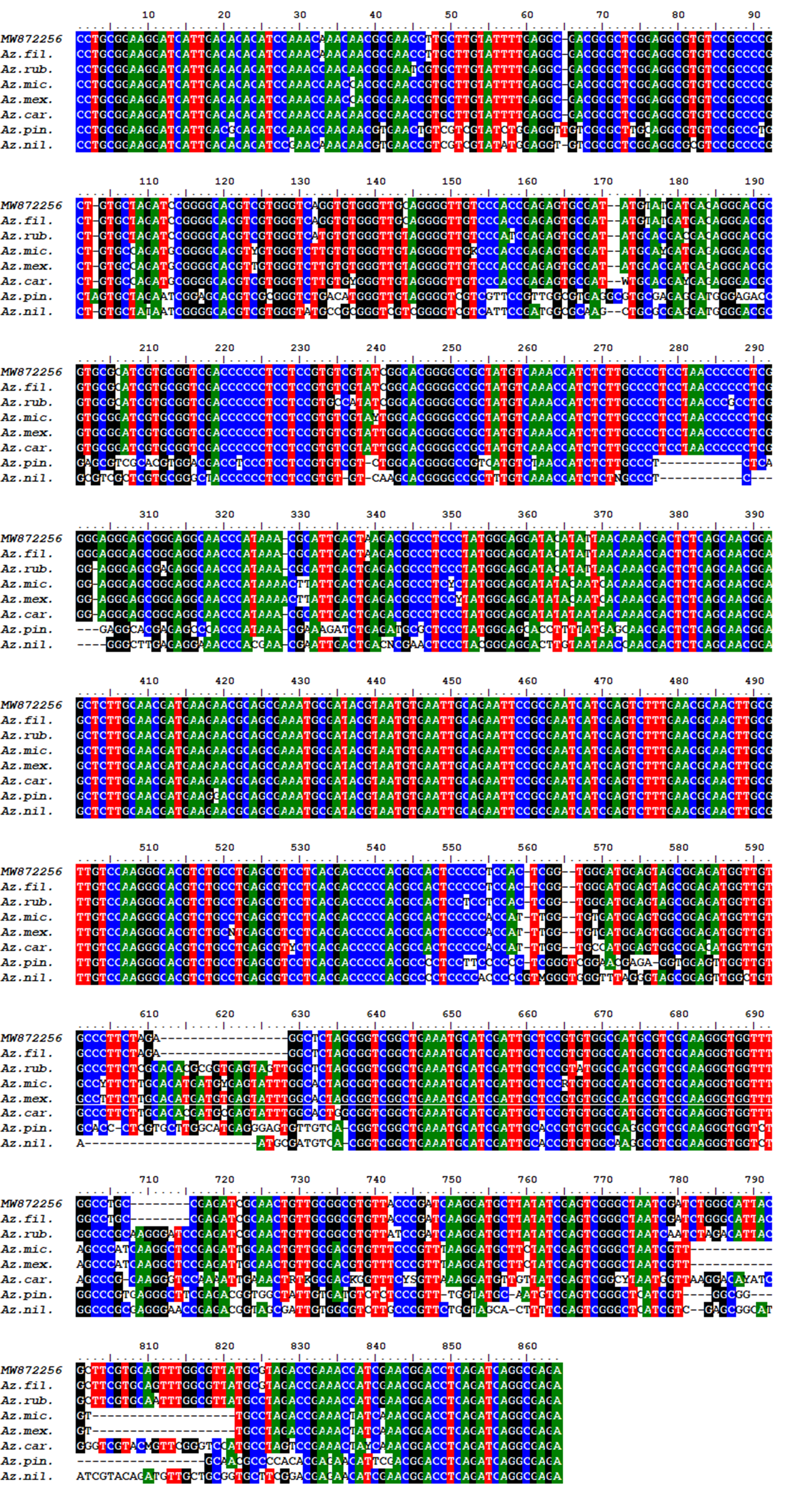

Supplement: Supplementary Figure S4 — Alignment of nuclear ribosomal DNA (rDNA) internal transcribed spacer (ITS) from Azolla spp. MW872256 (A. filiculoides collected from BG-PG); A. filiculoides (JX297314); A. rubra (DQ066492); A. microphylla (DQ066487); A. mexicana (DQ066477); A. caroliniana (DQ066473); A. pinnata (DQ066490); A. nilotica (DQ066470). [file Image_4.PNG]

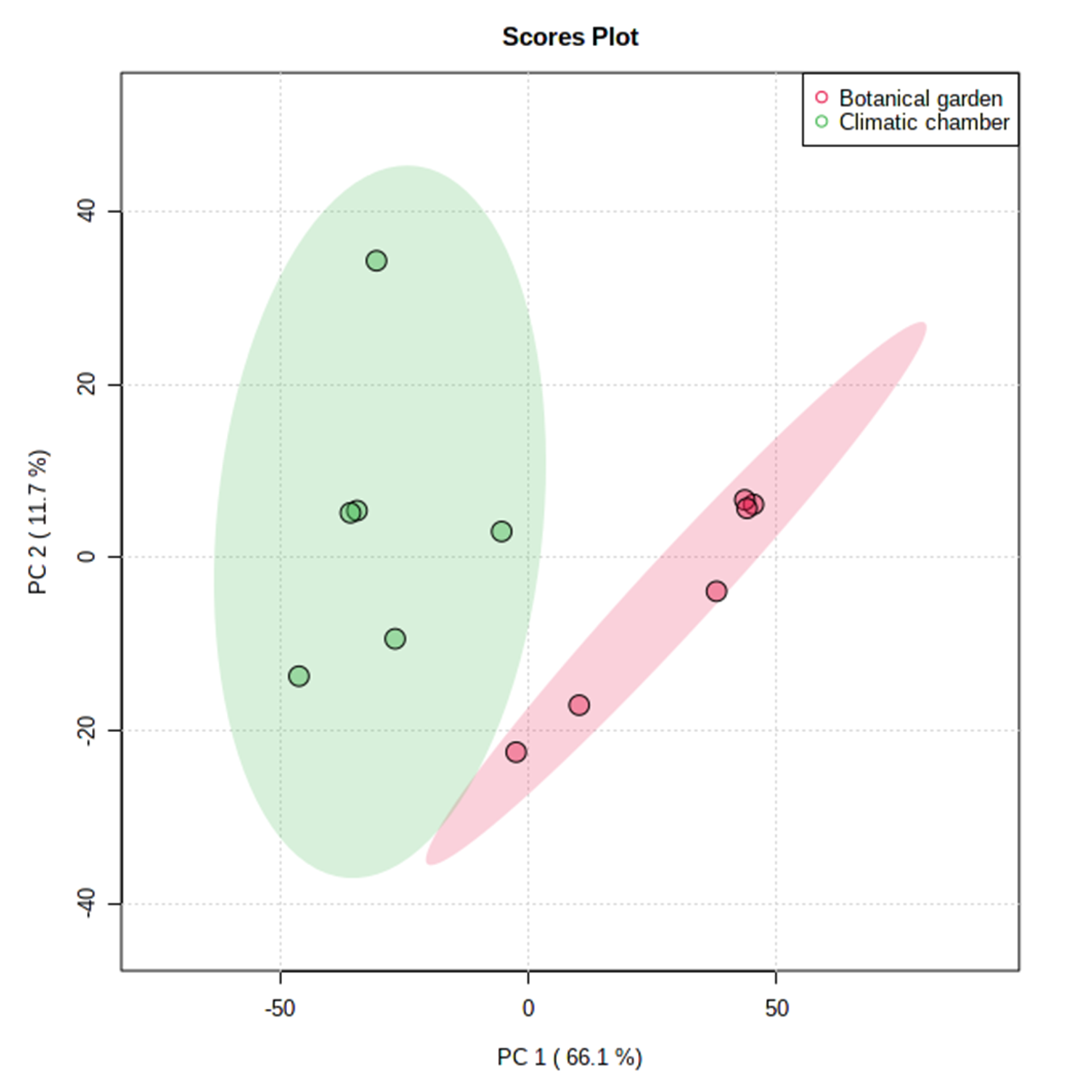

Supplement: Supplementary Figure S5 — Principal component analysis (PCA) score plot of the 146 polyphenols annotated from the six replicated samples for each growing condition tested in the CC vs. BG comparison. Total quantitative variances were clustered to reveal differences and relative similarities of different samples. 95% confidence regions (Hotelling's T2 eclipse) are displayed for each class. [file Image_5.PNG]

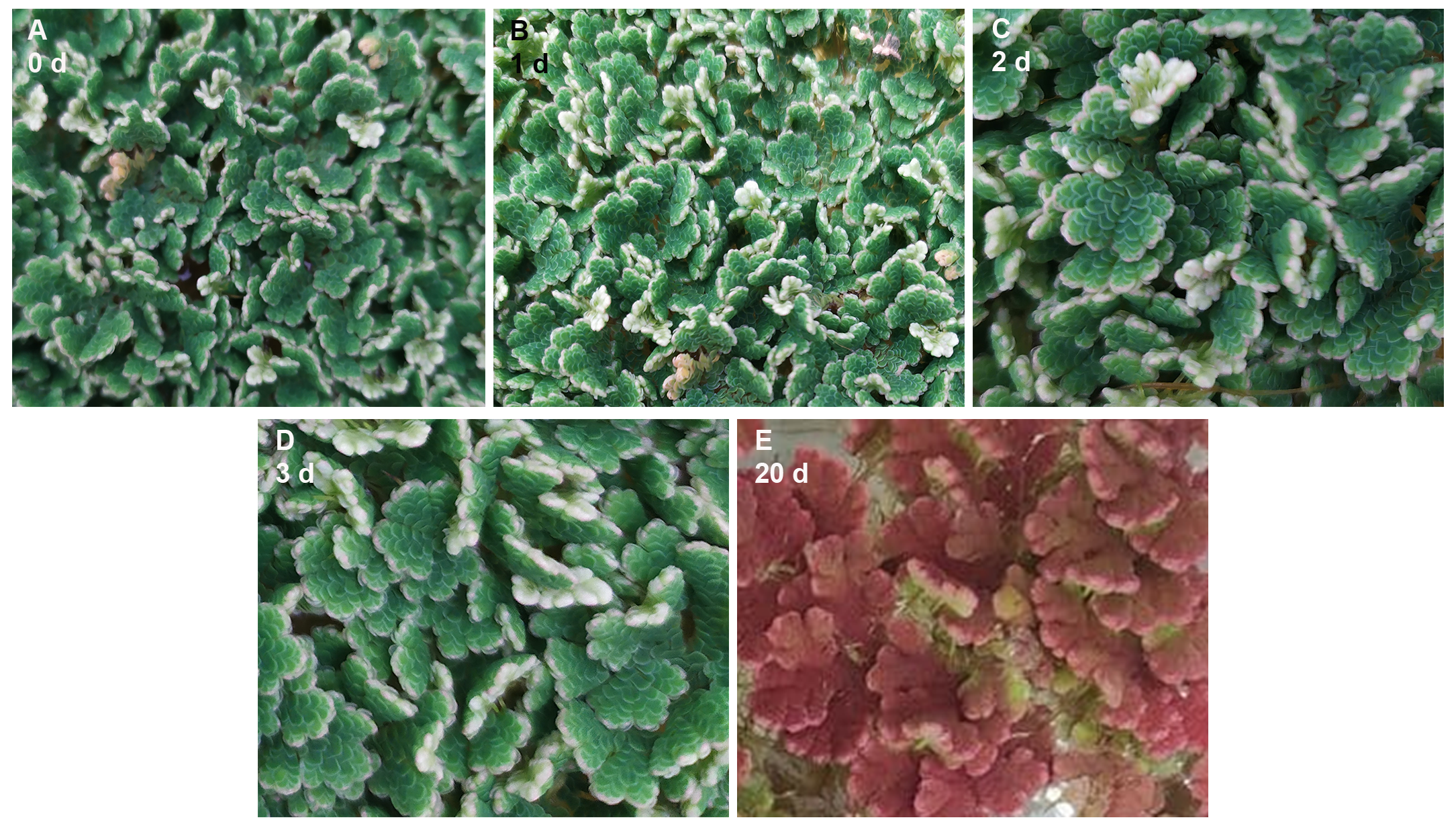

Supplement: Supplementary Figure S6 — Change in the color of Azolla fronds during the cold stress treatment under controlled conditions; on days (A) 0, (B) 1, (C) 2, (D) 3, and after (E) 20 days of treatment. [file Image_6.TIF]

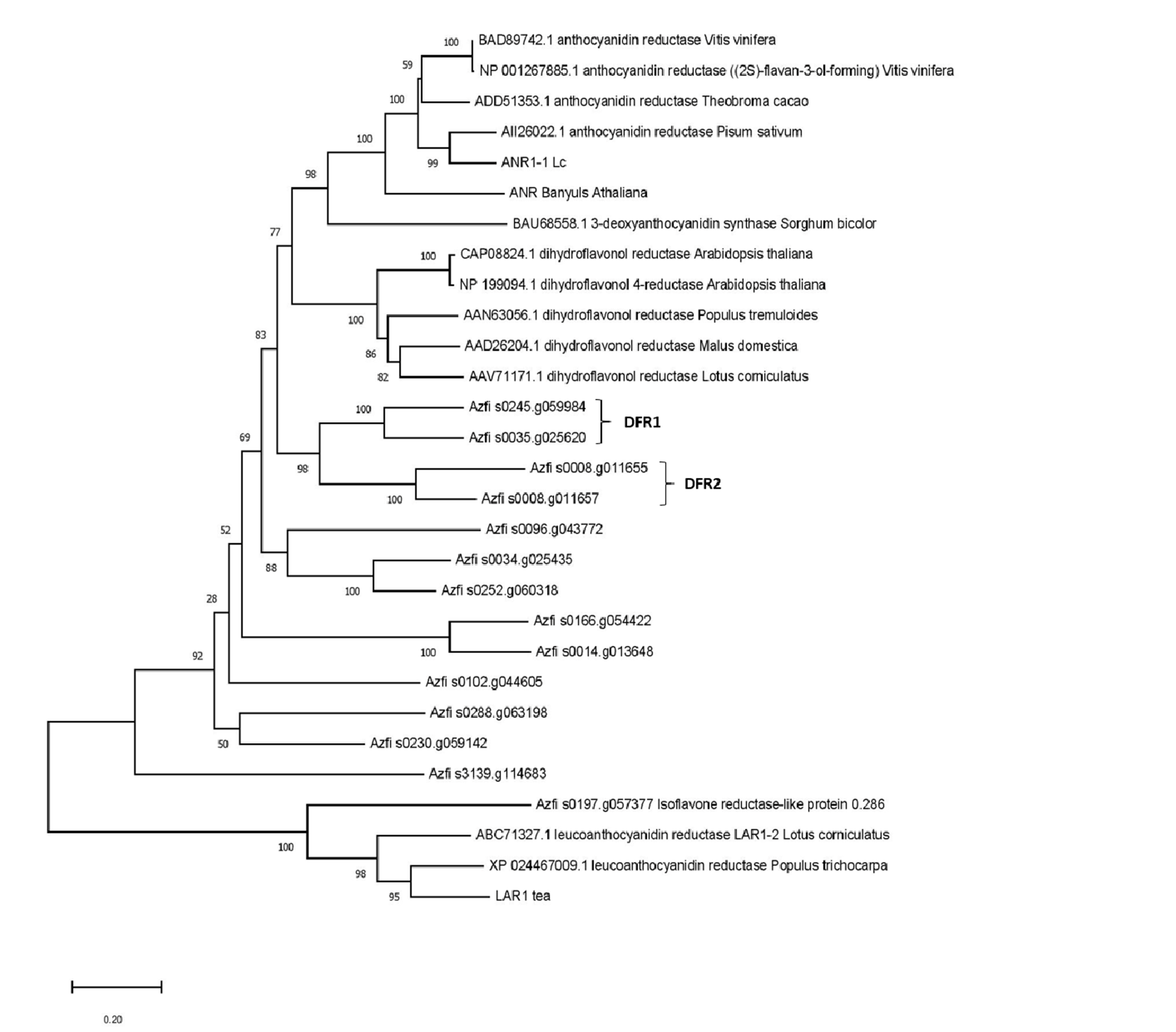

Supplement: Supplementary Figure S7 — Neighbor-joining tree of dihydroflavonol 4-reductase (DFR) proteins. The four DFR coding genes considered in this study are marked. The tree was built using 1,000 bootstraps. [file Image_7.PNG]
