# Supplementary material for: Light and Temperature Shape the Phenylpropanoid Profile of Azolla filiculoides Fronds
Source: Front Plant Sci. 2021 Oct 21;12:727667. doi: 10.3389/fpls.2021.727667 (PMC8567065; doi:10.3389/fpls.2021.727667)
Supplement: Supplementary Table S5 — Phenylpropanoids present in the time-course experiment but absent in the CC vs. BG comparison. [file Table_5.DOCX]

| Table S5 – Phenylpropanoids present in the time course experiment but absent in the CC *vs*. BG comparison |  |  |
| --- | --- | --- |
| **SUB-CLASS** | **#** | **PROPOSED COMPOUNDS** |
|  |  |  |
| **Hydroxycinnamic acids** | | |
|  | 1 | trans-3-Coumaric acid |
|  | 2 | (E)-N-2-Phenylethylcinnamamide |
|  | 3 | Cynarin |
| **Quinic acid and derivatives** |  |  |
|  | 4 | 3,5-Dicaffeoylquinic acid |
| **Chlorogenic acid and derivatives** |  |  |
|  | 5 | Chlorogenic acid |
|  | 6 | Chlorogenic acid, methyl ester |
|  | 7 | Isochlorogenic acid b |
| **Coumarins** |  |  |
|  | 8 | 6-Hydroxy-7-methoxycoumarin |
|  | 9 | 7-Hydroxy-3-(4-methoxyphenyl)-4-phenylcoumarin |
|  | 10 | 7,8-Dihydroxy-4-methylcoumarin |
| **Chalcones and derivatives** |  |  |
|  | 11 | Naringin dihydrochalcone |
| **Dihydrochalcones** |  |  |
|  | 12 | 3,2'-Dihydroxychalcone |
| **Flavanones** |  |  |
|  | 13 | Naringenin-4'-*O*-β-D-glucuronide |
|  | 14 | Naringenin-7-*O*-glucoside |
|  | 15 | Neoliquiritin |
|  | 16 | Glucoliquiritin apioside |
|  | 17 | Eriodictyol-7-*O*-glucoside |
|  | 18 | Narirutin 4'-*O*-glucoside |
| **Isoflavones** |  |  |
|  | 19 | 6''-*O*-Acetylgenistin |
| **Flavones** |  |  |
|  | 20 | Apigenin 7-glucoside |
|  | 21 | Apigenin 7-glucuronide |
|  | 22 | Apigenin-4'-glucoside |
|  | 23 | Luteolin 7-glucoside |
|  | 24 | 2''-Galloylhyperin |
|  | 25 | 3,3',7,8-Tetramethoxyflavone |
|  | 26 | 3',5'-Dimethoxy-3,5,7,4'-tetrahydroxyflavone |
|  | 27 | Licoflavone A |
| **Flavonols** |  |  |
|  | 28 | 3-*O*-[β-D-Apiofuranosyl-(1->2)-[β D-glucopyranosyl-(1->6)]-β-D-glucopyranoside]spinacetin |
|  | 29 | Hyperin |
|  | 30 | Kaempferol 3-ss-D-galactoside |
| **Flavan 3-ols** |  |  |
|  | 31 | (-)-Catechin |
| **Hydrolyzable tannins** |  |  |
|  | 32 | 1,2,4,6-Tetragalloylglucose |
| **Lignans** |  |  |
|  | 33 | (3S,3'S,4S,4'S)-7,7',9,9'-Tetramethoxy-3,3'-dimethyl-3,3',4,4'-tetrahydro-1H,1'H-[5,5'-bibenzo[g]isochromene]-4,4',10,10'-tetrol |
|  | 34 | Enterodiol |
|  | 35 | Silychristin |
| **Stilbenoids** |  |  |
|  | 36 | Piceatannol |
